# A Novel Artificial Electric Field Algorithm for Solving Global Optimization and Real-World Engineering Problems

**DOI:** 10.3390/biomimetics9030186

**Published:** 2024-03-19

**Authors:** Abdelazim G. Hussien, Adrian Pop, Sumit Kumar, Fatma A. Hashim, Gang Hu

**Affiliations:** 1 Department of Computer and Information Science, Linköping University, 581 83 Linköping, Sweden; adrian.pop@liu.se; 2 Faculty of Science, Fayoum University, Faiyum 63514, Egypt; 3 Australian Maritime College, College of Sciences and Engineering, University of Tasmania, Launceston 7248, Australia; sumit21sep1990@gmail.com; 4 Faculty of Engineering, Helwan University, Cairo 11795, Egypt; fatma_hashim@h-eng.helwan.edu.eg; 5 MEU Research Unit, Middle East University, Amman 11831, Jordan; 6 Department of Applied Mathematics, Xi’an University of Technology, Xi’an 710054, China; hugang@xaut.edu.cn

**Keywords:** artificial electric field algorithm, AEFA, escaping local operator, global optimization

## Abstract

The Artificial Electric Field Algorithm (AEFA) stands out as a physics-inspired metaheuristic, drawing inspiration from Coulomb’s law and electrostatic force; however, while AEFA has demonstrated efficacy, it can face challenges such as convergence issues and suboptimal solutions, especially in high-dimensional problems. To overcome these challenges, this paper introduces a modified version of AEFA, named mAEFA, which leverages the capabilities of Lévy flights, simulated annealing, and the Adaptive *s*-best Mutation and Natural Survivor Method (NSM) mechanisms. While Lévy flights enhance exploration potential and simulated annealing improves search exploitation, the Adaptive *s*-best Mutation and Natural Survivor Method (NSM) mechanisms are employed to add more diversity. The integration of these mechanisms in AEFA aims to expand its search space, enhance exploration potential, avoid local optima, and achieve improved performance, robustness, and a more equitable equilibrium between local intensification and global diversification. In this study, a comprehensive assessment of mAEFA is carried out, employing a combination of quantitative and qualitative measures, on a diverse range of 29 intricate CEC’17 constraint benchmarks that exhibit different characteristics. The practical compatibility of the proposed mAEFA is evaluated on five engineering benchmark problems derived from the civil, mechanical, and industrial engineering domains. Results from the mAEFA algorithm are compared with those from seven recently introduced metaheuristic algorithms using widely adopted statistical metrics. The mAEFA algorithm outperforms the LCA algorithm in all 29 CEC’17 test functions with 100% superiority and shows better results than SAO, GOA, CHIO, PSO, GSA, and AEFA in 96.6%, 96.6%, 93.1%, 86.2%, 82.8%, and 58.6% of test cases, respectively. In three out of five engineering design problems, mAEFA outperforms all the compared algorithms, securing second place in the remaining two problems. Results across all optimization problems highlight the effectiveness and robustness of mAEFA compared to baseline metaheuristics. The suggested enhancements in AEFA have proven effective, establishing competitiveness in diverse optimization problems.

## 1. Introduction

Metaheuristic optimization algorithms have recently garnered significant attention and become a focal point for many computer science and engineering researchers. They are increasingly prevalent across various domains, including numerical optimization [1,2], cloud computing [3], neural networks [4], feature selection [5,6], classification [7], clustering [8], predicting chemical activities [9], text document clustering [10], and face detection & recognition [11].

Examples of metaheuristic algorithms include Genetic Algorithms (GA) [12], CMAES) [13], Cuckoo Search Optimization (CS) [14], the Harmony Search (HS) Algorithm [15], Artificial Bee Colony (ABC) [16], the Krill Herd Algorithm (KHA) [17], the Ant Lion Optimizer (ALO) [18], Moth-flame Optimization (MFO) [19], the Crow search Algorithm (CSA) [20], the Whale Optimizer Algorithm (WOA) [21], the Lightning Search Algorithm (LSA) [22], the Salp Swarm Algorithm (SSA) [23], Harris Hawks Optimization (HHO) [24], Virus Colony Search (VCS) [25], and the Snake Optimizer (SO) [26].

Metaheuristics (MHs) typically adopt strategies from swarm intelligence (SI), evolutionary algorithms (EA), physics-based principles, and human-based concepts. EAs are inspired by biological behaviors, while SI draws on food foraging, mutation, territorial fights, and mating behaviors. Physics laws and human behavior also influence optimizer development [27]. Consequently, these refined algorithms are increasingly applied to effectively address challenges in engineering design optimization, yielding more promising solutions.

Despite the wide range of problem-solving capabilities offered by metaheuristics, the “No free Lunch” hypothesis suggests that no single MH can provide the best solution for every complex problem [28]. Each optimization problem requires a specific strategy to be effectively addressed [29].

While solving complex engineering problems, metaheuristics may have drawbacks such as slow convergence and being trapped in local search domains, resulting in higher computational costs [30]. To address these limitations, researchers have devised hybridized, modified, and enhanced MHs that incorporate more beneficial attributes. A few examples include the Hybrid Grey Wolf and Crow Search [31], Hybrid Heat Transfer and Passing Vehicle Search [32], Hybrid Artificial Hummingbird-Simulated Annealing [33], Modified Symbiotic Organisms Search [34], Modified Marine Predator Algorithm [35], Improved Ant Colony Optimization [36], and Improved Salp Swarm Algorithm [37]. To create effective MHs, a balance between global diversification and local intensification is crucial. Both exploration and exploitation phases are important in finding superior solutions and achieving results in the least amount of time. Despite numerous hybrid MHs being implemented in engineering design optimization over the last few decades, the quest for even more potent methods is ongoing. This field continues to evolve and presents new challenges for researchers to address.

The Artificial Electric Field Algorithm (AEFA) is a recent population-based algorithm proposed by Anita and Yadav [38] that is inspired by the electrostatic force of Coulomb’s law. AEFA has been successfully employed in many applications such as photovoltaics [39], fuel cell estimation [40], the economic load dispatch problem [41], non-linear system modeling [42], the quadratic assignment problem [43], soil shear [44], and wind turbine allocation [45].

In addition, many researchers have introduced different variants of AEFA algorithms. For example, Izci et al. [46] hybridized AEFA with the Nelder–Mead simplex. Likewise, Petwal and Rani [47] developed a multiobjective version of AEFA. They strengthened AEFA by adding polynomial mutation and bounded exponential crossover operators. A novel set of velocity and position bounds has been proposed by Anita et al. [48] to address engineering problems. Anita and Yadav [49] developed a discrete AEFA for high-order graph matching. Furthermore, a detailed study of stability and exploratory abilities was presented in [50].

Kahraman et al. [51] introduce a novel approach called the Natural Survivor Method (NSM), devised as a model for population management mirroring natural processes, taking into account environmental variables and analytical relationships. Within the NSM framework, scores reflecting individuals’ adaptability to their natural surroundings are computed to identify survivors. Additionally, the update mechanism in this proposed method is constructed based on NSM scores rather than traditional fitness values.

Although AEFA has demonstrated effectiveness in addressing intricate engineering problems, it exhibits a notable susceptibility to converging toward suboptimal solutions. The iterative process of population size selection poses challenges, as optimal sizes vary across different problems. Like any other algorithm, AEFA demonstrates a moderate convergence rate, potentially requiring more time than alternative algorithms to reach an optimal solution. Moreover, the efficacy of AEFA is notably contingent on parameter choices, including the number of particles, electric charge, and inertia. AEFA performance may be limited on large-scale problems due to high computational complexity. Moreover, AEFA may face premature convergence issues, particularly in multimodal optimization problems. Furthermore, if the algorithm focuses too much on exploration, it may sacrifice exploitation, and vice versa, thus striking a balance between global diversification and local intensification is crucial for optimizing performance. Hybridizing AEFA with other algorithms or the inclusion of performance improvement strategies can result in faster convergence, higher precision, and greater robustness [52]. As AEFA is a relatively new optimizer, there is great potential for uncovering new improvements that can further enhance its efficiency and performance.

The objective of this research was to enhance the performance of the existing AEFA algorithm by addressing its limitations, with a focus on achieving faster convergence, increased accuracy, and improved robustness. To achieve these goals, a modified version of AEFA named modified AEFA (mAEFA) is introduced based on two techniques: Lévy flights and Simulated Annealing (SA). The first operator is used to increase randomness and the second is used to increase exploitation.

This study offers the following significant contributions:

- The Lévy flight distribution mechanism is incorporated to increase the search space of AEFA and improve the exploration potential of the algorithm. This integration aims to prevent the algorithm from getting trapped in local optima, thereby contributing to an overall improvement in performance;
- A simulated annealing mechanism is integrated with AEFA to improve search exploitation by allowing the algorithm to accept solutions that are worse than the current best solution with a certain probability, which helps to avoid getting stuck in local optima and explore other areas of the search space. This can lead to finding better solutions that may have been missed otherwise. Therefore, hybridizing simulated annealing into an algorithm can lead to improved performance and robustness;
- A thorough evaluation of mAEFA performance, utilizing both quantitative and qualitative methods, is carried out on a variety of complex CEC 2017 constraint benchmarks with varying characteristics;
- The behavior of mAEFA is evaluated and compared to 12 prevalent MH approaches on five engineering benchmark problems drawn from diverse fields.

The rest of this paper is structured as follows: Section 2 discusses recent work on metaheuristics and Section 3 introduces the original AEFA. Section 4 presents the suggested modified version of AEFA. Section 5 details the results of the conducted statistical tests and respective discussion, and Section 6 concludes the paper, including outlining future directions.

## 2. Related Work

The Lévy flight (LF) mechanism is a popular choice for researchers aiming to bolster the optimization efficacy of algorithms, spanning diverse research domains such as control system design [53], wind speed forecasting [54], and high-dimensional optimization problems [55]. Characterized by random steps, the application of LF enables optimization algorithms to navigate the vicinity of existing solutions, while intermittent significant leaps mitigate the risk of entrapment within local minima. For instance, Zhang et al. [56] recently demonstrated that LF enhances particle diversity within PSO, thereby refining the accuracy of lithium-ion battery State-of-Health prediction. Similarly, Hussien et al. [57] noted the efficacy of LF-based Transient Search Optimization in augmenting the transient response of terminal voltages within islanded microgrids. Furthermore, Barua et al. [58] amalgamated the LF strategy with the Arithmetic Optimization Algorithm to address various engineering optimization challenges. Their findings underscore the hybrid algorithm’s superior optimization capabilities, requiring fewer evaluations and surpassing several established algorithms in performance benchmarks.

Simulated annealing (SA) is an MH extensively employed to enhance the search capability of optimization algorithms [59]. Distinguished by its probabilistic jump feature, inspired by the physical process of annealing solids, SA effectively mitigates the risk of stagnation in local optima, facilitating the attainment of global optima. For example, Xu et al. [60] endeavored to enhance the Whale Optimization Algorithm by integrating it with SA, yielding superior optimization performance and stability across multiple dimensional problems compared to alternative algorithms such as WOA, GWO, and PSO. Similarly, Fontes et al. [61] employed a hybrid of SA and PSO for tackling the job shop scheduling problem, achieving high-quality solutions within reasonable computation times. In another study, Sajjad et al. [62] noted that the incorporation of SA notably enhanced the convergence of the multiobjective Grasshopper Algorithm when applied to problem solving in IoT applications.

The performance of population-based metaheuristic algorithms is significantly influenced by the size, nature, and diversity of the initial population, as well as the number of iterations. Agushaka et al. [63] observed that BA performed better with larger population sizes, while GWO, WOA, BOA, MS, and LSHADE-cnEpSin benefited from more iterations. Conversely, MFO, LSHADE, EHO, and HHO showed optimal performance with balanced population sizes and iterations. Kazimipour et al. [64] categorize population initialization techniques based on randomness, compositionality, and generality aspects. While random number generators are commonly used for initialization, their limitations have prompted researchers to explore alternative distributions, such as Latin Hypercube Sampling (LHS), to enhance efficiency [65]. Quasirandom sequences like Halton, Sobol, and Faure have demonstrated effectiveness in uniformly covering the search space, as seen in PSO and Global Best PSO. Chaotic sequences have also shown promise in initializing populations in CS and BFO, despite concerns about computational complexity. Hybridization with other metaheuristic algorithms, like the Greedy Randomized Adaptive Search Procedure (GRASP) and Metropolis–Hastings (MH), has the potential for improving initialization schemes, though scalability and time complexity are challenges. Leveraging ad hoc knowledge of the problem domain, as seen in the Bat Algorithm (BA), and innovative approaches such as patch environments and quasi-opposition-based learning in CS, aim to improve algorithm efficacy. Each method presents unique advantages and challenges, highlighting the importance of selecting suitable initialization strategies tailored to the problem domain and available computational resources [63,64,65].

The MH process involves two main steps: selection of solution candidates from the population and determination of the search direction. Selection methods are vital in MHs and are categorized into three types: non-deterministic, deterministic, and probabilistic [66]. Non-deterministic methods randomly select solution candidates to enhance search diversity. Deterministic methods consider fitness values, selecting the best candidates to guide the search towards successful positions. Probabilistic methods combine characteristics of both elitist and random methods, with examples including the roulette wheel and tournament methods. New selection methods, such as Fitness–Distance balance (FDB) [66], Fitness–Distance-Constraint (FDC) [67], Adaptive FDB [68], and Dynamic FDB [69], address specific challenges in MH search.

## 3. Artificial Electric Field Algorithm

The Artificial Electric Field Algorithm (AEFA) is a recent population-based metaheuristic algorithm that mimics Coulomb’s electrostatic force law [38]. Coulomb’s electrostatic law states that the force between any two charged particles is inversely proportional to the distance squared between the particles and directly proportional to their charge product.

Particles can migrate/move in the search space. With the help of the electrostatic force, particles are able to interact with each other (either repulsion or attraction).

In AEFA, one only considers the attraction force, which means that particle with the highest charge will absorb other particles with lower charges. The objective function in AEFA is the candidate solution (particle) charges and the agent fitness value. To mathematically model the electric force, the following steps are used.

The position of *i*-th agent $\left(X_{i}\right)$ can be given as $X_{i}=\left(x_{i}^{1},x_{i}^{2},...,x_{i}^{d}\right)$, where $x_{i}^{d}$ is the position of i-th agent at d-dimension.

The position of the best agent (solution) obtained by any particle, *i*, at any time, *t*, denoted as $P_{i}\left(t\right)$, is given by the following Equation (1)

(1) $$P_{i}^{d}\left(t+1\right)=\left\{\begin{array}{cc} P_{i}^{d}\left(t\right) & f\left(P_{i}\left(t\right)\right)<f\left(X_{i}\left(t+1\right)\right)\\ X_{i}^{d}\left(t+1\right) & \mathrm{if}\;f\left(X_{i}\left(t+1\right)\right)<f\left(P_{i}\left(t\right)\right) \end{array}\right.$$

where $P_{i}$ is the position of the personal best particle. $X_{best}$ is the position of the best charged particle determined based on fitness. The best objective value via all particles is given as $P_{best}=X_{best}$.

To calculate the total force that is acting on agent *i* at time *t*, the following equation is used

(2) $$F_{ij}^{d}=\sum_{j=1,j\neq i}^{N}rand\left(\right)F_{ij}^{d}$$

where rand() is a random number between 0 and 1, *N* refers to the number of search agents, and $F_{i}$ refers to resultant force that affect on the *i*-th agent.

The acceleration of the *i*-th agent at *d* dimension can be calculated using Newton’s second law of motion as below

(3) $$a_{i}^{d}\left(t\right)=\frac{Q_{i}E_{i}^{d}\left(t\right)}{M_{i}\left(t\right)}$$

$Q_{i}$ and $E_{i}$ represent the charges and electric field of the $i^{th}$ particle, and $M_{i}\left(t\right)$ refers to $i^{th}$ particle mass. The velocity and position of particles can be updated using the two following equations:

(4) $$V_{i}^{d}\left(t+1\right)=rand\left(\right)\times V_{i}^{d}\left(t\right)+a_{i}^{d}\left(t\right)$$

(5) $$X_{i}^{d}\left(t+1\right)=X_{i}^{d}\left(t\right)+V_{i}^{d}\left(t+1\right)$$

where rand() is a random number belongs to the interval [0,1]. The particles’ charge can be obtained using fitness functions and assuming that each particle has the same charge.

## 4. Proposed Algorithm

**Figure 1:**
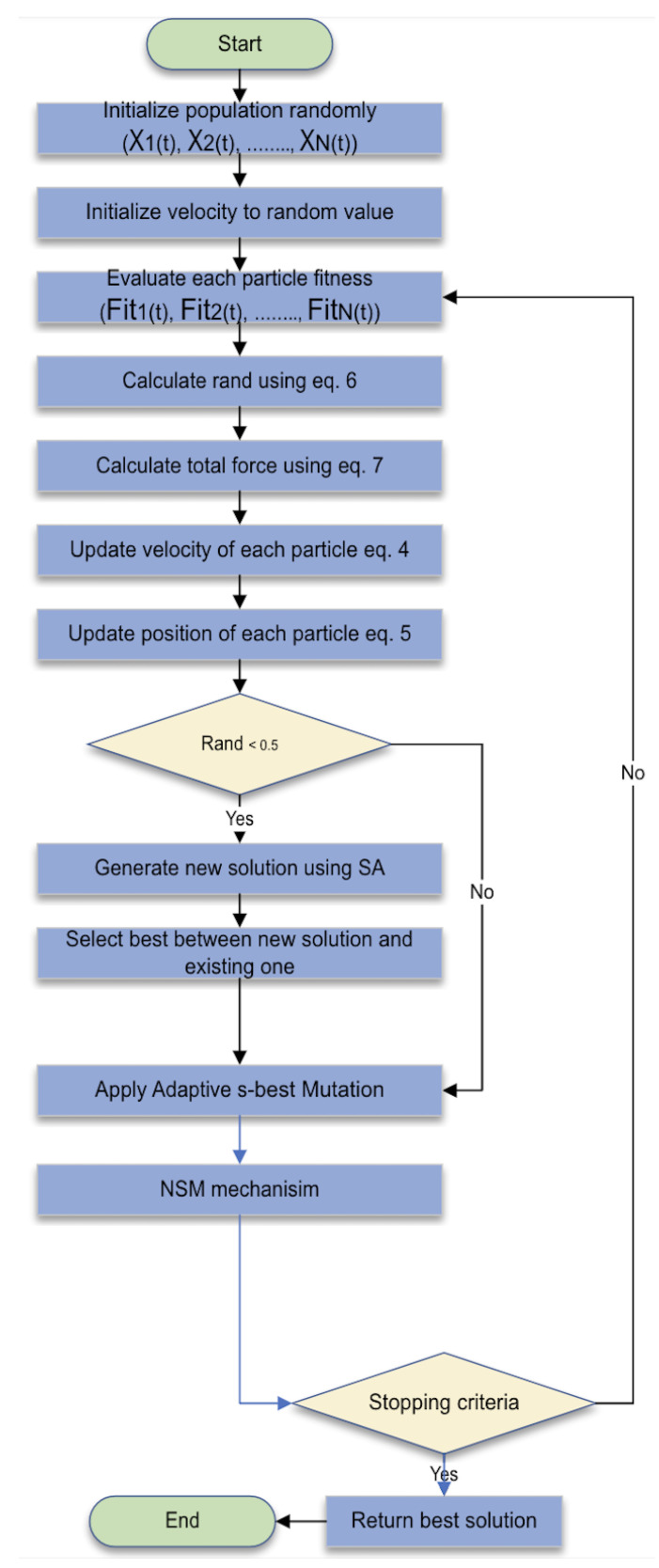
mAEFA flow chart.

Despite the success and the power of AEFA, it has many drawbacks, as it may become stuck in local optima or have slow convergence, especially in high-dimensional and complex problems. This paper suggests an improved version of AEFA called modified AEFA (mAEFA) that tries to overcome the limitation of the original algorithm using two techniques, Lévy flights and Simulated Annealing, in each updating process. The flow chart is given in Figure 1.

### 4.1. Lévy Flights

Randomization has a great emphasis and effect in algorithms as it prevents them from falling in local optimal regions/areas. The Lévy flight mechanism has been successfully implemented and employed in many algorithms. In this version, we used Lévy flight to generate a random vector instead of the vector given in Equation (2) and use it to calculate the force as follows:

(6) ran=2×rand−Lévy(D)

(7) $$F_{i}=\sum_{j=1,j\neq i}^{N}ranF_{ij}^{d}$$

where rand is a random number between 0 and 1 and *D* is the problem dimension. This will provide the search agent with a high opportunity to cover the entire search space.

### 4.2. Simulated Annealing

Simulated Annealing (SA) is a single-solution metaheuristic algorithm developed by Kirkpatrick et al. [70] that can be considered as an extension of the hill climbing technique. SA use a certain probability in accepting a worse solution to be able to avoid local optima. SA use Boltzmann probability, $P=e^{-\theta /T}$, where θ is the difference between solutions and *T* refers to a temperature parameter.

SA is employed here to enhance the exploitation abilities of the AEFA. In this paper, we used SA to generate a new solution and update the current agent if the new solution is better than the current one. Here, *T* is equal to 2×|N|, where *N* is the number of agents.

### 4.3. Adaptive *S*-Best Mutation

The equilibrium between exploration and exploitation stands out as a crucial aspect determining an algorithm’s effectiveness in search capability. In the original AEFA algorithm, the absence of a gradual transition from exploration to exploitation results in new solutions being generated from the current solution without any discernible bias. To address this limitation, we introduce the *s*-best mutation approach, where S represents the top-ranked solution based on its fitness. This mutation strategy randomly selects one solution from the top S candidates to generate new solutions. The range of potential solutions is controlled by variables S1,S2,⋯,SN, and S0,⋯1. Through this approach, each new solution, indexed as i, is derived by either mutating the current solution or randomly mutating any solution selected from the top *S* candidates. The parameter *S* decreases non-linearly over time, enabling a broad exploration of the search space initially, gradually shifting towards a focus on solutions closer to the global optimum. This straightforward approach enhances diversity at the onset of generations, facilitating effective exploration, while transitioning towards exploitation as generations progress. During the exploitation phase, the algorithm concentrates on a narrower range of solutions surrounding the global best. The *s*-best mutation scheme operates as follows:

(8) $$y=X_{sbest}+F\times \left(X_{r1}-X_{r2}\right)$$

where $X_{best}$ signifies the agent derived from the top S×N agents, differing from the deterministic best solution. Additionally, the individuals $X_{r1}$ and $X_{r2}$ represent two distinct agents randomly selected from the entire generation, meeting the criteria that $r_{1}\neq r_{2}$, and neither is equal to the current agent. *F* denotes a constant and real-value factor typically chosen to be between 0 and 1, and *r* is linearly decreased as specified as follows:

(9) $$S_{t}=1-\left(1-\frac{1}{N}\right)\times \frac{t-1}{T-1}$$

where *T* signifies the maximum iteration number, *t* represents the current time, and *N* denotes the size of the population. Therefore, larger values of S are associated with earlier solutions, facilitating effective exploration. Conversely, as generations progress, *S* diminishes to foster exploitation enhancements.

### 4.4. NSM Operator

The survivor-selection process based on NSM scores operates according to a model that analytically connects the three described criteria. According to this model, the solution candidate that contributes more to the diversity of the mating pool and population compared to its competitors, while also achieving a better fitness value for the objective function, will survive. Within the NSM framework, the survivor is determined as the one that obtains the best score according to these three criteria.

## 5. Experimental Results and Discussion

To evaluate the proposed mAEFA method, this study employs the CEC’17 test suite, consisting of 30 functions commonly used to assess MHs [71]. Five engineering problems are also used for evaluation. The objective is to assess the search capability and convergence behavior of the proposed method. Given the stochastic nature of MHs, the experiments are conducted 30 times to account for randomness and observe result variations across runs.

Several newly developed algorithms are employed for comparison with the suggested mAEFA. These algorithms consist of the Coronavirus Herd Immunity Optimizer (CHIO) [72], Gravitational Search Algorithm (GSA) [73], Smell Agent Optimization (SAO) [74], Grasshopper Optimisation Algorithm (GOA) [75], Particle Swarm Optimization (PSO) [76], Liver Cancer Algorithm (LCA) [77], and basic AEFA [38]. For fairness in comparing algorithms, all were executed on the same hardware system to solve the CEC’17 test suite. The experiments were standardized with a maximum of 500 iterations. MATLAB 2021 served as the programming language for all algorithms, operating on a 64-bit Windows 8.1 system with an i7 Core and 8 GB RAM.

### 5.1. Experimental Series 1: CEC’17

#### 5.1.1. Comparing mAEFA with State-of-Art Algorithms

CEC’17 includes functions representing diverse, complex, and dynamic optimization problems, commonly used for evaluating algorithm effectiveness. This study assesses the mAEFA algorithm using these functions, gaining insights into its effectiveness. Furthermore, it’s worth noting that each experiment is constrained by a maximum evaluation limit of 50,000 iterations. The setup entails employing 30 agents within a 30-dimensional space, as delineated in Table 1. Throughout the entirety of the experimentation, MATLAB2021 is utilized as the programming language, operating on a 64-bit Windows 8.1 platform. Table 2 presents the 30 functions, classified into four sets: F1 to F3 represents the unimodal set, F4 to F10 represents the multimodal set, F11 to F20 represents the hybrid set, and F21 to F30 represents the composition set. F2 is excluded from the evaluation process, leaving a total of 29 functions utilized to evaluate the mAEFA algorithm and other algorithms. The search range for all test functions, as indicated in Table 2, spans from −100 to 100, with a dimensionality of 30. In order to test and validate the enhanced version of mAEFA, we conducted a comparison with the original AEFA as well as six other distinct algorithms: CHIO, GSA, SAO, GOA, PSO, and LCA. The parameter seetinbg of each algorithm is given in Table 3.

The evaluation was based on the calculation of the average and standard deviation.

The comparison results are presented in Table 4. It is evident from the table that the developed algorithm (mAEFA) ranks first in 20 out of the 29 functions. Additionally, mAEFA obtains the second-best results in four functions and the third-best result in three functions. In contrast, GSA ranks first in only five functions, while AEFA achieves the best results in only four functions. Among the majority of the CEC’17 29 test functions, the algorithms that exhibit the lowest performance are SAO, GOA, LCA, and CHIO.

To validate the effectiveness of mAEFA, the statistical results of the nonparametric Wilcoxon rank-sum (WRS) test are presented in Table 5. This test helps determine the significance of the differences between mAEFA and other algorithms measured with a *p*-value of ≤0.05. Based on the results, the mAEFA algorithm demonstrates superior performance compared to the LCA algorithm across all 29 test functions. It also outperforms SAO and GOA in 28 functions, CHIO in 27 functions, PSO in 25 functions, GSA in 24 functions, and AEFA in 17 functions, showcasing better results in each case.

**Figure 2:**
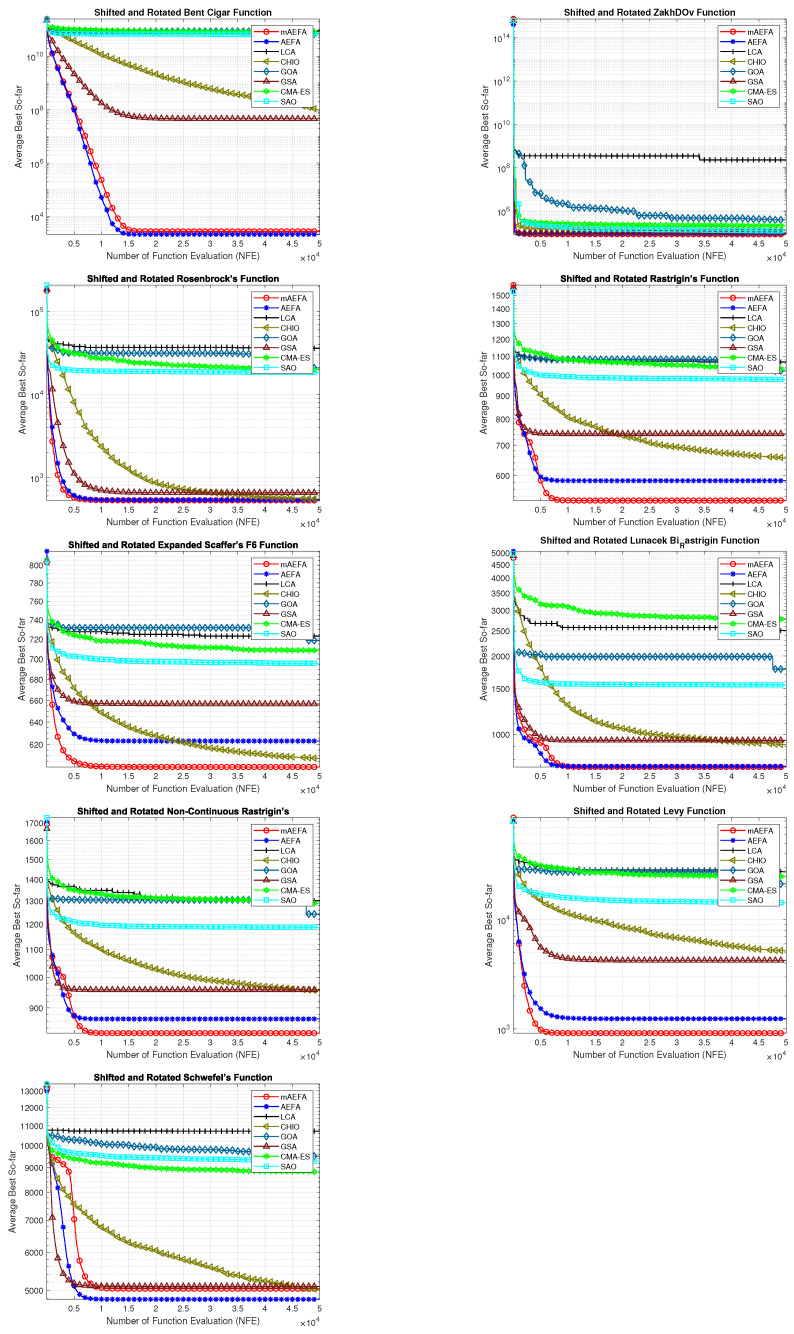
Graphical representation of convergence curves for functions ranging from F1 to F10 using CEC’17 with a dimensionality of 30.

**Figure 3:**
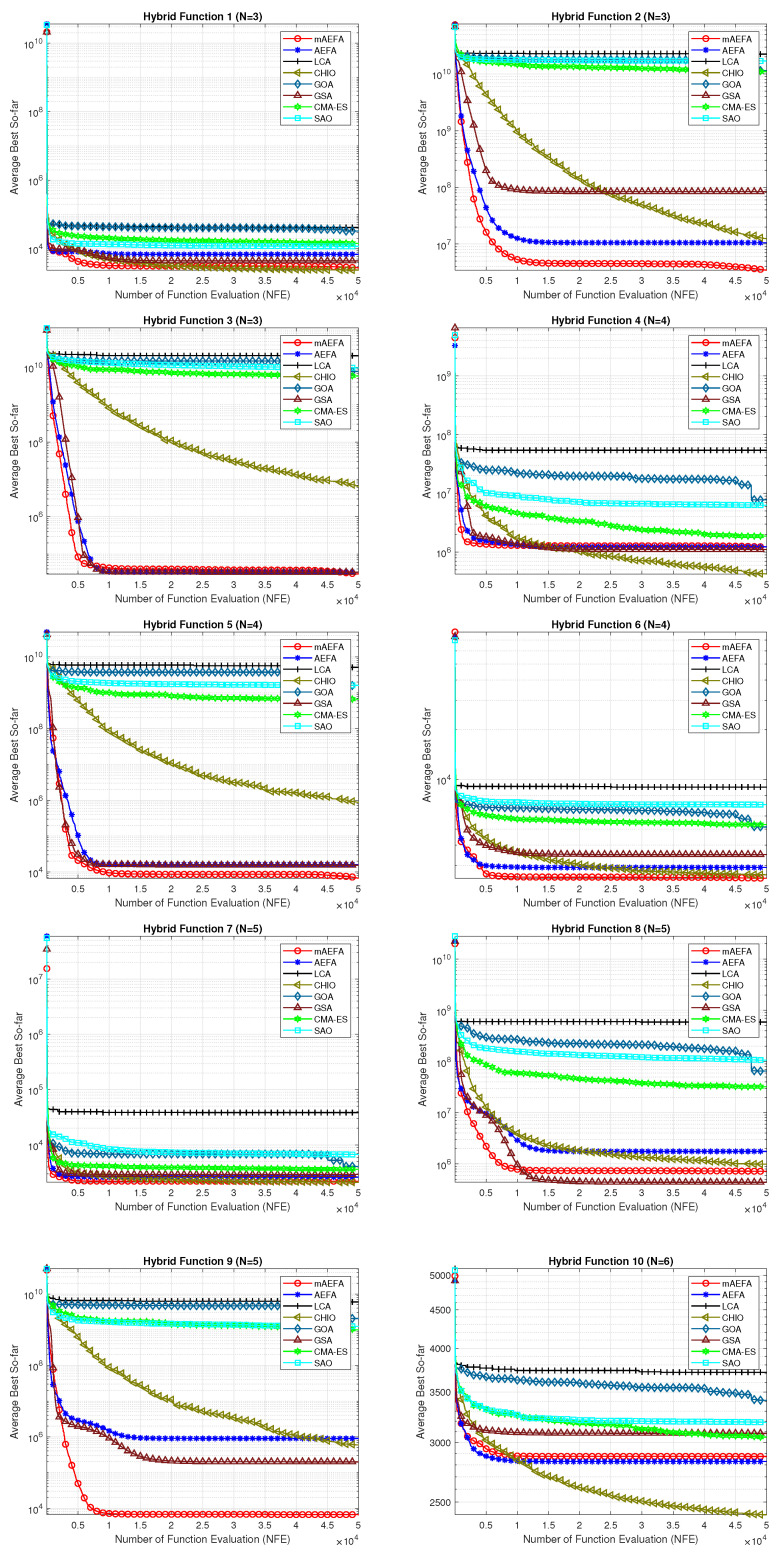
Graphical representation of convergence curves for functions ranging from F11 to F20 using CEC’17 with a dimensionality of 30.

**Figure 4:**
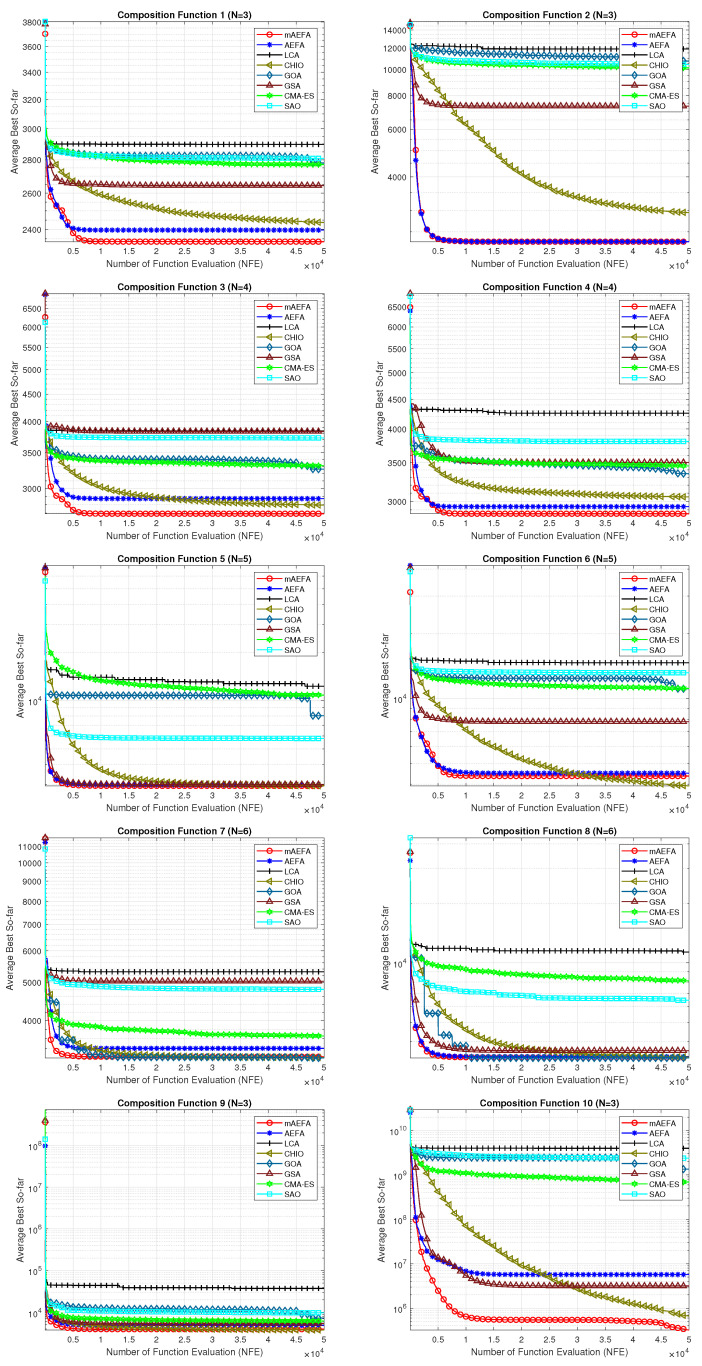
Graphical representation of convergence curves for functions ranging from F21 to F30 using CEC’17 with a dimensionality of 30.

The convergence behavior of the proposed mAEFA algorithm, along with the other compared algorithms, applied to the CEC’17 functions is depicted in Figure 2, Figure 3 and Figure 4. It can be observed that mAEFA demonstrates rapid convergence for F3–F6, F8, F11–F14, F16, F17, F19, F21, F23, F24, and F27–30 compared to the classical AEFA algorithm. Additionally, mAEFA on average depicted better convergence behavior relative to the other algorithms in most of the CEC test suite benchmarks. The mAEFA algorithm demonstrates a balanced exploration–exploitation trade-off, evident in its convergence plots, showcasing both faster convergence and the discovery of optimal solutions during the search process.

**Figure 5:**
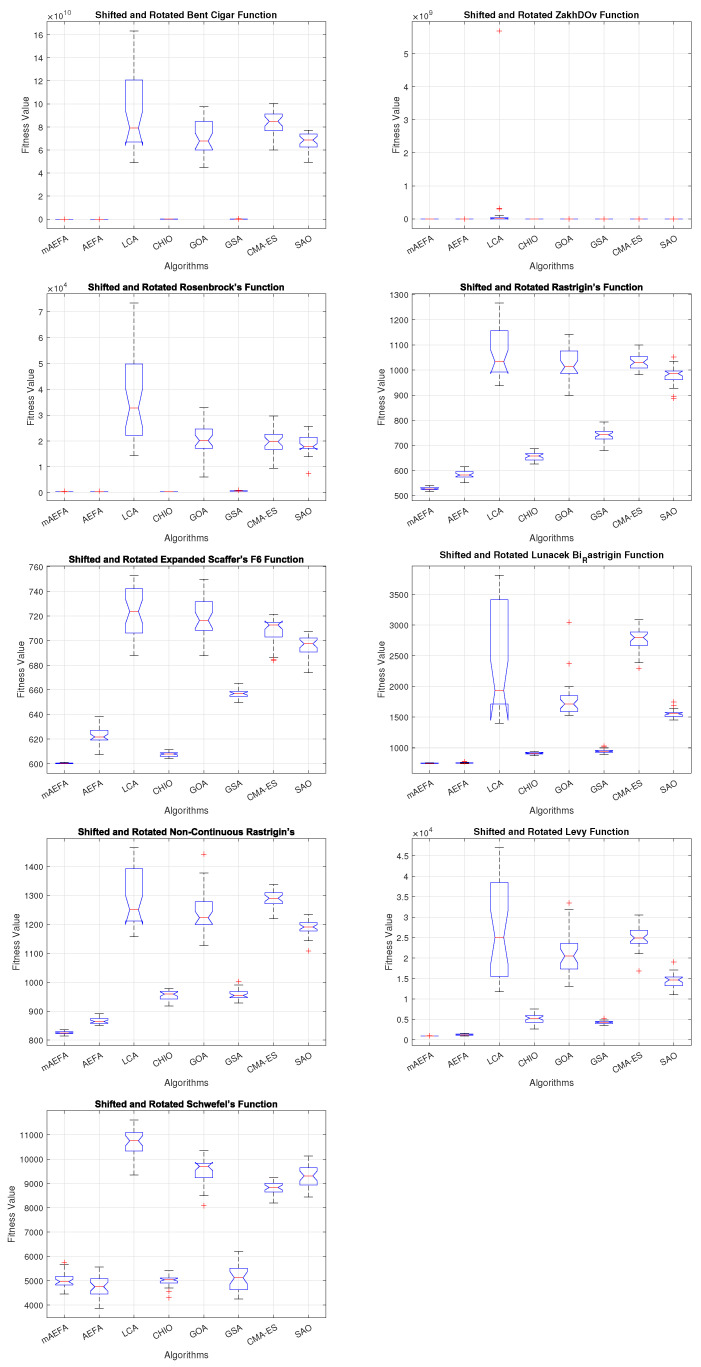
Box plots illustrating the distribution of results for functions spanning from F1 to F10 using CEC’17 with a dimensionality of 30.

**Figure 6:**
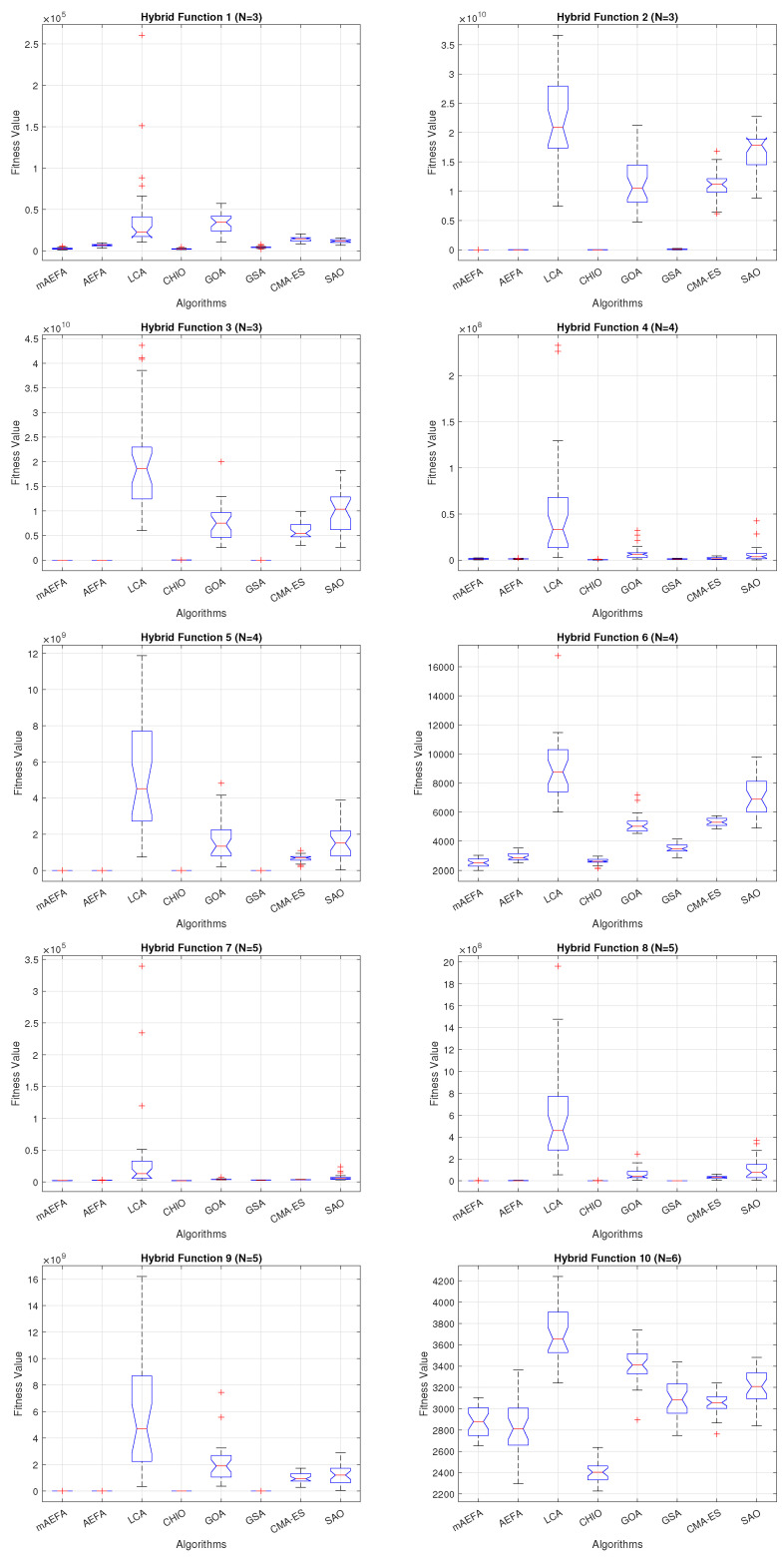
Box plots illustrating the distribution of results for functions spanning from F11 to F20 using CEC’17 with a dimensionality of 30.

**Figure 7:**
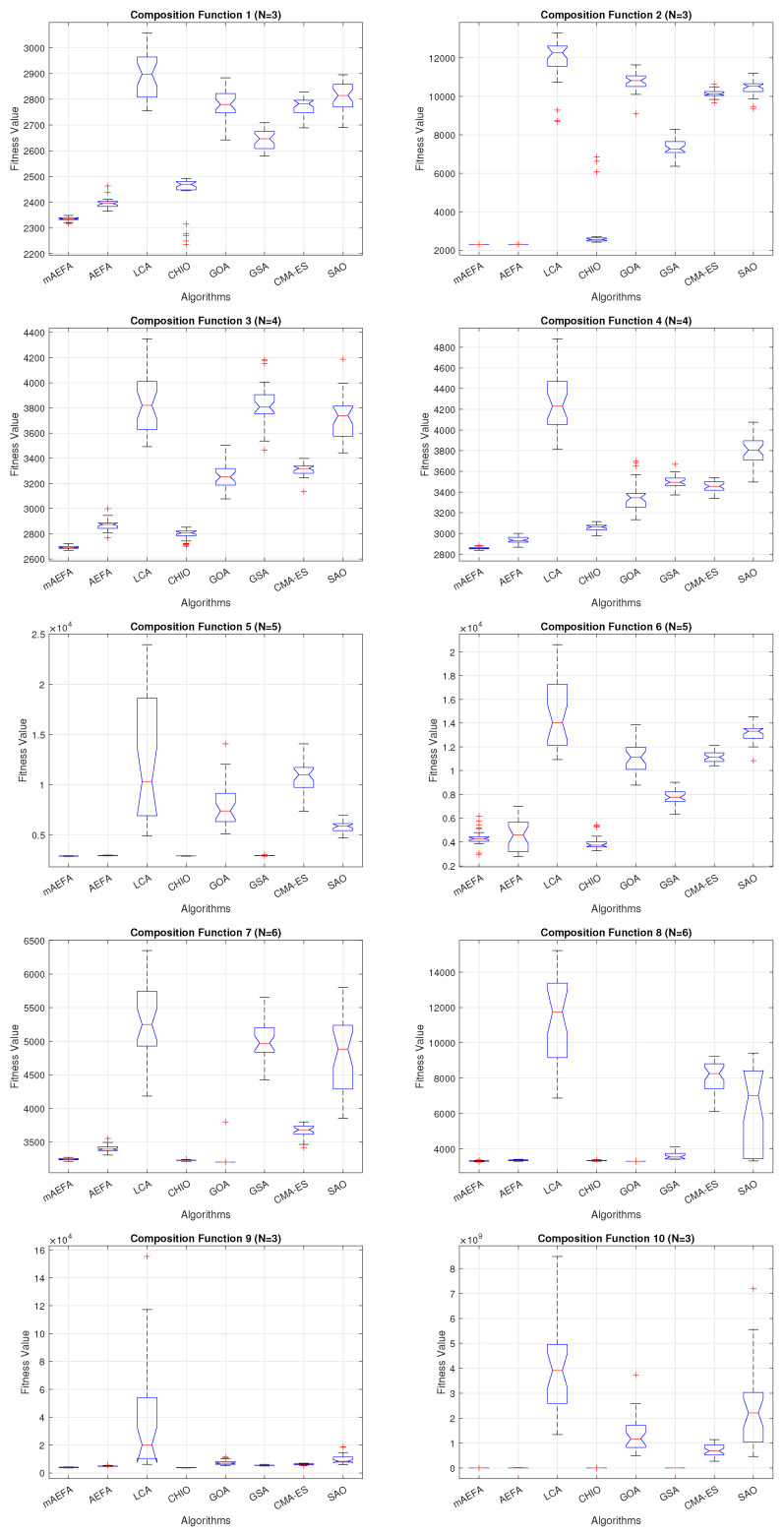
Box plots illustrating the distribution of results for functions spanning from F21 to F30 using CEC’17 with a dimensionality of 30.

Additionally, Figure 5, Figure 6 and Figure 7 shows box plots for the proposed algorithm and the other compared algorithms, highlighting the distribution of results and the degree of proximity and similarity across multiple runs. The box plot is utilized to represent the minimum, maximum, and mean values for each algorithm. Box plots serve as an effective visual representation for presenting the performance values obtained in the 1st, 2nd, and 3rd] quarters of the experiment. A horizontal line within the box denotes the median value, while the whiskers extending outside indicate the variability beyond the upper and lower quartiles. Ideally, smaller box sizes and lower variability are preferred. Figure 5, Figure 6 and Figure 7 illustrate box plots for mAEFA for the majority of the functions, displaying narrow boxes with the lowest median and variability.

#### 5.1.2. Comparing mAEFA with Fitness–Distance Balance Variants

In this subsection we compare mAEFA with two other FDB variants, namely FDB-SOS [66] and dfDB-MRFO [69]. Table 6 presents the statistical analysis results in terms of minimum, maximum, average, and standard deviation values, along with the ranks of the algorithms. Upon examination, mAEFA consistently outperforms the other variants across several functions, as evidenced by its lower minimum and average values, as well as its higher ranks in most cases. For example, in functions *F*5–*F*9, F19, *F*21–*F*24, and *F*26, mAEFA demonstrates superior performance with lower minimum and average values compared to dfDB-MRFO and FDB-SOS, resulting in higher ranks. Additionally, mAEFA shows competitive performance in other functions, such as F1, F10, F15, F27, and F29, where it achieves comparable results to the other variants. Overall, the comparative analysis suggests that mAEFA exhibits favorable performance across a range of benchmark functions compared to dfDB-MRFO and FDB-SOS. These findings underscore the efficacy of mAEFA as a competitive algorithm for solving optimization problems, particularly in the context of the functions analyzed in this study.

### 5.2. Experimental Series 2: Engineering Problems

In this section, we explore five distinct engineering challenges to assess the effectiveness of the proposed solution in dealing with problems that involve constraints. The following constraint problems, which are commonly encountered and widely utilized, have been considered: speed reducer design (SRD), pressure vessel design (PVD), cantilever beam design (CBD) and the multi-product batch plant (MPBP) problem, and Industrial Refrigeration System Optimal Design.

#### 5.2.1. Speed Reducer Design Problem

This problem involves optimization with the objective of minimizing the weights associated with various design elements. The optimization process incorporates constraints related to gear teeth, stress, deflection ratios of bending, surface, and shafts [78]. The SRD is based on seven design variables ($z_{1},z_{2},z_{3},z_{4},z_{5},z_{6},\;\mathrm{and}\;z_{7}$) that aim to minimize the weight. These variables represent face width, teeth module, pinion teeth number, the length of the first shaft between the bearings, the length of the second shaft between the bearings, and the diameters of the first and second shafts.

**Figure 8:**
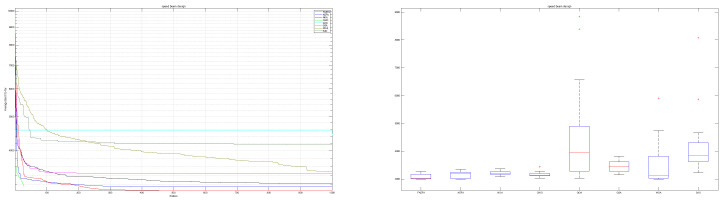
Speed reducer design.

The mAEFA algorithm attains the top rank with a significantly low standard deviation, as indicated by the results in Table 7. Furthermore, Table 8 illustrates its competitive performance across all seven variables, underscoring the noteworthy differentiation of mAEFA in comparison to alternative algorithms. Figure 8 illustrates a distinct divergence in convergence patterns among the algorithms, where mAEFA exhibits superior convergence by attaining exceptionally low values in the final iteration of the search process.

(10) $$\begin{array}{cc} \;\mathrm{Let} & \\ \;\mathrm{Min}\; & f\left(z\right)=0.7854z_{1}z_{2}^{2}\left(3.3333z_{3}^{2}+14.9334z_{3}-43.0934\right)\\ & -1.508z_{1}\left(z_{6}^{2}+z_{7}^{2}\right)+7.4777\left(z_{6}^{3}+z_{7}^{3}\right)+0.7854\left(z_{4}z_{6}^{2}+z_{5}z_{7}^{2}\right)\\ \;\mathrm{Subject}\;\mathrm{to}:\; & R_{1}\left(z\right)=\frac{27}{z_{1}z_{2}^{2}z_{3}}-1\leq 0\\ \; & R_{2}\left(z\right)=\frac{397.5}{z_{1}z_{2}^{2}z_{3}}-1\leq 0\\ \; & R_{3}\left(z\right)=\frac{1.93z_{4}^{3}}{z_{2}z_{3}z_{6}^{4}}-1\\ \; & R_{4}\left(z\right)=1.93z_{5}^{3}z_{2}z_{3}z_{7}^{4}-1\leq 0\\ \; & R_{5}\left(z\right)=\frac{1}{110z_{6}^{3}}\sqrt{{\left(\frac{745z_{4}}{z_{2}z_{3}}\right)}^{2}+16.9\times 10^{6}}-1\leq 0\\ \; & R_{6}\left(z\right)=\frac{1}{85z_{7}^{3}}\sqrt{{\left(\frac{745z_{5}}{z_{2}z_{3}}\right)}^{2}+157.5\times 10^{6}}-1\leq 0\\ \; & R_{7}\left(z\right)=\frac{z_{2}z_{3}}{40}-1\leq 0\\ \; & R_{8}\left(z\right)=\frac{5z_{2}}{z_{1}}-1\leq 0\\ \; & R_{9}\left(z\right)=\frac{z_{1}}{12z_{2}}-1\leq 0\\ \; & R_{1}0\left(z\right)=\frac{1.5z_{6}+1.9}{z_{4}}-1\leq 0\\ \; & R_{1}1\left(z\right)=\frac{1.1z_{7}+1.9}{z_{5}}-1\leq 0\\ \;\mathrm{where}\; & 2.6\leq z_{1}\leq 3.6,0.7\leq z_{2}\leq 0.8,17\leq z_{3}\leq 28,7.3\leq \\ \; & x_{4}\leq 8.3,7.8\leq z_{5}\leq 8.3,2.9\leq z_{6}\leq 3.9,\;and\;5\leq z_{7}\leq 5.5 \end{array}$$

#### 5.2.2. Pressure Vessel Design Problem

The PVD problem, introduced by Kannan and Kramer [79], is the second engineering design problem in this study, assessing the effectiveness of the proposed mAEFA. This problem revolves around optimizing the cost associated with PVD, aiming to minimize it. The cost is determined by four design variables: $z_{1},z_{2},z_{3},\;\mathrm{and}\;z_{4}$, which represent shell thickness, head thickness, inner radius, and cylinder length, respectively. Hashim et al. [80] provide an extensive description of the mathematical model for this problem. The PVD problem’s mathematical model can be defined using the following set of equations.

**Figure 9:**
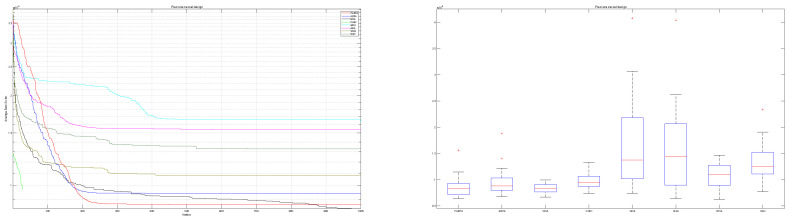
Pressure vessel design problem.

According to the findings presented in Table 9, the mAEFA algorithm obtains the first rank with a minimum mean value of 6412.152. Figure 9 demonstrates comparable behavior across all algorithms, with mAEFA consistently achieving minimum values during the later iterations. Table 10 presents the variable values obtained by each algorithm for this specific problem. Notably, mAEFA achieves competitive values for $z_{1}$ and $z_{3}$, and it secures the second-lowest value for $z_{2}$. Table 10 clearly indicates that mAEFA surpasses all algorithms, except for the LCA algorithm, indicating a substantial difference in its performance.

(11) $$\begin{array}{cc} \;\mathrm{Let} & \\ \;\mathrm{Min}\; & f\left(z\right)=0.6224z_{1}z_{3}z_{4}+1.7781z_{2}z_{3}^{2}+3.1661z_{1}^{2}z_{4}+19.84z_{1}^{2}z_{3}\\ \;\mathrm{Subject}\;\mathrm{to}:\; & R_{1}\left(z\right)=-z_{1}+0.0193z\\ \; & R_{2}\left(z\right)=-z_{2}+0.00954z_{3}\leq 0\\ \; & R_{3}\left(z\right)=-\pi z_{3}^{2}z_{4}-\left(4/3\right)\pi z_{3}^{3}+1,296,000\leq 0\\ \; & R_{4}\left(z\right)=z_{4}-240\leq 0\\ \;\mathrm{where}\; & 0\leq z_{i}\leq 100,i=1,2\\ \; & 10\leq z_{i}\leq 200,i=3,4 \end{array}$$

#### 5.2.3. Multi-Product Batch Plant Problem

In the MPBP problem, the production process begins after the customer submits their order, with each order representing a specific product. Throughout production, the batch size for each order remains constant. Each order is assigned due dates and release dates. At each stage, there are dedicated processing units that exclusively operate within that stage. The objective of this problem is to minimize the makespan, considering additional constraints such as unallowed unit assignments, order due dates, release dates, and storage issues. The problem’s formulation is delineated by Equations (12)–(23), with Equation (12) addressing the constraint related to order assignment, ensuring that each order (*i*) can only be processed on a single unit (*j*) at a specific step (*s*).

(12) $$\sum_{j\in J_{is}}Z_{ij}=1,\;i\in I_{j}$$

Equations (13)–(15) reveal unit order sequencing, with Equations (13) and (15) indicating a singular first order for each unit *j*, and Equation (12) depicting sequence constraints for different orders *i* and *i*′ on the same unit *j*.

(13) $$\sum_{i\in I_{j}}ZF_{ij}\leq 1$$

(14) $$\sum_{i^{\prime }\in I_{s}}X_{i^{\prime }is}+\sum_{j\in J_{is}}Z_{ij}=1,\;I\in I_{s}$$

(15) $$Z_{ij}\geq ZF_{ij},\;i\in I_{j}^{\#}$$

Equations (16) and (17) represent unit assignment constraints. When integer variables $X_{ii^{\prime }s}$ or $X_{i^{\prime }s}$ are activated, it implies that orders $i^{\prime }$ and *i* must be processed on the same unit *j*.

(16) $$2\left(X_{ii^{\prime }s}+X_{i^{\prime }s}\right)+\sum_{j\in J_{is}-J_{i^{\prime }s}}Z_{ij}+\sum_{j\in J_{I^{\prime }s-J_{is}}Z_{i^{\prime }j}}Z_{ij}\leq 2,\;i^{\prime }>i,\left(i^{\prime },i\right)\in I_{s}$$

(17) $$Z_{ij}\leq Z_{i^{\prime }j},\;j\in J_{is}\cap J_{i^{\prime }j},\left(i^{\prime },i\right)\in I_{s}$$

Equations (16)–(19) represent order timing constraints. Equation (18) pertains to timing constraints within one order’s various steps, while Equation (19) addresses timing constraints for multiple orders on the same unit. Additionally, when considering unit release time $UR_{j}$ or order release time $OR_{i}$, Equations (20) and (21) come into play. Equation (22) is applicable for cases involving due date $DD_{i}$.

(18) $$T_{is^{\prime }}\geq T_{is}+\sum_{j\in J_{is}}Z_{ij}PT_{ijs^{\prime }}=n_{sis},\;s\in S_{i}$$

(19) $$M\left(1-X_{ii^{\prime }s}\right)+T_{i^{\prime }s}\geq T_{is}+\sum_{j\in J_{is}}Z_{ij}PT_{ijs^{\prime }}=n_{sis},\;s\in S_{i}$$

(20) $$T_{is}\geq \sum_{j\in J_{is}}ZF_{ij}UR_{j},\;i\in I_{s}$$

(21) $$T_{is}\geq OR_{i},\;s=fs_{i}$$

(22) $$T_{is}+\sum_{j\in J_{is}}Z_{ij}t_{ij}\leq DD_{i},\;i\in I,s=Is_{i}$$

The goal is to minimize the makespan, and the objective function is defined as follows:

(23) $$\mathrm{Make}-\mathrm{span}=\min\left(\max\left(T_{is}+\sum_{j\in J_{is}}Z_{ij}PT_{ij}\right)\right),\;s=Is_{i}$$

Meeting all constraints is straightforward except for Equation (22) when minimizing the makespan with metaheuristic algorithms. To accommodate Equation (22), a penalty function is employed, and Equations (24) and (25) are utilized to calculate the objective function in this study:

(24) $$d_{i}\geq \max\left(T_{is}+\sum_{j\in J_{is}}Z_{ij}PT_{ij}-DD_{i}\right),\;i\in I,s=Is_{i}$$

(25) $$\mathrm{Objective}\;\mathrm{function}=\min\left(\max\left(T_{is}+\sum_{j\in J_{is}}Z_{ij}PT_{ij}\right)\right)+M\sum_{i\in I}d_{i},s=Is_{i}$$

Equation (24) defines the penalty function, which is employed to penalize violations in Equation (25). When the completion time of each order exceeds its respective due date, Equation (24) comes into effect, leading to a reduction in the objective value in Equation (25).

**Figure 10:**
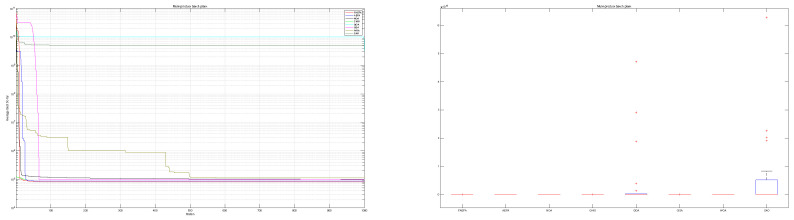
Multi-product batch plant problem.

According to the data presented in Table 11, the mAEFA algorithm secures the first position in terms of statistical ranking amongst all compared algorithms. Table 12 showcases that mAEFA attains competitive results across various problem variables. Figure 10 depicts that mAEFA, alongside some other algorithms, demonstrates similar convergence behavior reaching minimum values. However, both CHIO and SAO exhibit premature convergence behavior, indicating a tendency to get trapped in local minima.

#### 5.2.4. Industrial Refrigeration System Optimal Design

A refrigeration system utilizes coolant to decrease the temperature of a hot stream, going through three distinct phases. Each phase incorporates a heat exchanger on one side and a boiling cooler on the other. The pumping current is determined by the heat exchanger’s surface area. Moreover, the boiling temperature for the refrigerant is established at the start of each phase. Designing an efficient cooling system entails calculating the surface areas of the three surfaces of the liquid cooling heat exchanger.

The refrigeration system is designed to dissipate 4186.8 J/kg°C of heat while pumping a flow rate of 10,800 kg per hour from an initial temperature of 10 °C to −55 °C. Operating for a minimum of 300 days per year, the refrigeration system’s key parameters include a refrigerant latent heat (λ) of 232.600 J/Kg and an overall heat transfer coefficient of 1130 J/s m^2^ °C. The primary design objective is to minimize the cost of the three steps, as specified by Equation (26).

(26) $$Cost=\sum_{i=1}^{3}\left[c_{i}{\left(C_{i}\right)}^{0.5}+d_{i}M_{i}\right]$$

The optimization aims to obtain competitive values for design variables, including fluid temperatures, heat exchange area, and liquid refrigerant addition rates in each step, with a focus on minimizing costs. The optimization process places significant emphasis on the temperature of the liquid refrigerant in each step, described as follows:

(27) $${\mathrm{Temp}}_{1}=-18\;{}^{\circ}C,{\mathrm{Temp}}_{2}=-40\;{}^{\circ}C,{\mathrm{Temp}}_{3}=-62\;{}^{\circ}C$$

The temperature of the incoming fluid to the system is 10 °C, denoted as $Temp_{0}$, while the temperature of the outgoing fluid from the system is −55 °C, represented as $Temp_{3}$. It is essential for the output temperature at each step to be higher than the temperature of the refrigerant. As a result, the conditions for the design variables can be expressed as shown in Equations (28)–(33).

(28) $${\mathrm{Temp}}_{0}=10\;{}^{\circ}C\geq {\mathrm{Temp}}_{1}\geq -18\;{}^{\circ}C40TTC$$

(29) $${\mathrm{Temp}}_{1}\geq {\mathrm{Temp}}_{2}\geq -40\;{}^{\circ}C$$

(30) $$F_{i}=H_{i}\times C_{i}\times {\left(\Delta {\mathrm{Temp}}_{i}\right)}_{\ln}$$

The log mean temperature difference at stage *i* is:

(31) $${\left(\Delta {\mathrm{Temp}}_{i}\right)}_{\ln}=\frac{{\mathrm{Temp}}_{i-1}-{\mathrm{Temp}}_{i}}{\ln\left(\frac{{\mathrm{Temp}}_{i}-{\mathrm{Temp}}_{R_{i}}}{{\mathrm{Temp}}_{i-1}-{\mathrm{Temp}}_{R_{i}}}\right)}$$

The energy balance over refrigerant is:

(32) $$F_{i}=\lambda_{i}\times M_{i}$$

$\lambda_{i}$ is the penalty factor, where $F_{i}$ is the ratio of heat flow, J/s. The energy balance over the fluid is:

(33) $$F_{i}=V\times k_{l}\times \left({\mathrm{Temp}}_{i-1}-{\mathrm{Temp}}_{i}\right)$$

where Kl is the specific heat of fluid, J/kg°C and V is the hot fluid pump ratio, kg/hr.

**Figure 11:**
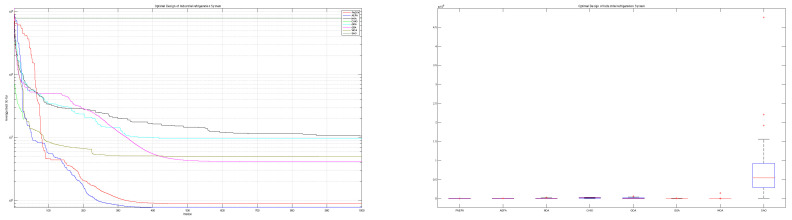
Optimal design of Industrial refrigeration system.

Based on the findings presented in Table 13, the proposed mAEFA algorithm achieves a competitive rank when compared to other algorithms. Additionally, as indicated in Table 14, mAEFA attains the smallest values for the majority of the problem variables and the best solution in comparison to other algorithms. Table 14 demonstrates that mAEFA yields significant results in comparison to all other algorithms. Convergence behavior, as depicted in Figure 11, varies among the algorithms in their approach towards the optimal solution. Notably, mAEFA demonstrates a favourable convergence pattern, reaching minimum values in later search iterations.

## 6. Conclusions and Future Work

The main contributions of this research work can be summarized as follows:

1. A modified mAEFA approach is developed by combining the original AEFA algorithm with Lévy flights, simulated annealing, and Adaptive *s*-best Mutation and Natural Survivor Method (NSM) mechanisms. The mAEFA utilizes Lévy flights distribution to enhance exploration and convergence, while the simulated annealing approach assists in avoiding local optima;
2. The performance of the mAEFA algorithm is scrutinized on various benchmark functions and engineering design problems. Comparative analysis with popular metaheuristics demonstrates the superiority and competitiveness of mAEFA;
3. In solving composite CEC’17 benchmark functions, mAEFA shows superior performance compared to other algorithms under comparison, except for specific functions. In unimodal, multimodal, and composite CEC’17 functions, mAEFA is either superior, equal, or ranked second among compared algorithms, with faster convergence observed;
4. Box plot analysis and Wilcoxon rank-sum test results further validate the superiority and robustness of mAEFA over comparative algorithms in CEC’17 test functions;
5. Performance testing of mAEFA on five engineering design problems demonstrates its superior capability to effectively solve practical optimization problems compared to other algorithms.

Nonetheless, similar to other metaheuristic algorithms, mAEFA faces limitations in solving all optimization problems across different domains, as highlighted by the No Free Lunch theorem. In future research, we aim to validate the effectiveness of the proposed mAEFA by testing it on more intricate continuous and discrete optimization problems. Moreover, there is potential to apply this algorithm to address various problems in diverse fields such as cloud task scheduling, image segmentation, engineering design, air quality prediction, finance, material science, and environmental sciences, among others.

In the future, a multiobjective version of the developed algorithm can be designed to tackle multiobjective problems. Furthermore, the authors intend to use the same combination to test other metaheuristic algorithms.

## Figures and Tables

**Table 1:** Experiments parameters settings.

No.	Parameter Name	Value
1	Population Size	30
2	Dim	30
3	Max number of evaluation	50000

**Table 2:** Benchmark functions.

No.	Types	Name	Opt
F1	Unimodal	Shifted and Rotated Bent Cugar Function	100
F2		Shifted and Rotated Sum of Different Power Function	200
F3		Shifted and Rotated Zakharov Function	300
F4	Multimodal	Shifted and Rotated Rosenbrock’s Function	400
F5		Shifted and Rotated Rastrigin’s Function	500
F6		Shifted and Rotated Expanded Scaffer’s F6 Fuction	600
F7		Shifted and Rotated Lunacek Bi-Rastrigin Function	700
F8		Shifted and Rotated Non-Continuous Rastrigin’s Function	800
F9		Shifted and Rotated Lécy Function	900
F10		Shifted and Rotated Schwefel’s Function	1000
F11	Hybrid	Hybrid function 1 (N = 3)	1100
F12		Hybrid function 2 (N = 3)	1200
F13		Hybrid function 3 (N = 3)	1300
F14		Hybrid function 4 (N = 4)	1400
F15		Hybrid function 5 (N = 4)	1500
F16		Hybrid function 6 (N = 4)	1600
F17		Hybrid function 6 (N = 5)	1700
F18		Hybrid function 6 (N = 5)	1800
F19		Hybrid function 6 (N = 5)	1900
F20		Hybrid function 6 (N = 6)	2000
F21	Composition	Composition Function 1 (N = 3)	2100
F22		Composition Function 2 (N = 3)	2200
F23		Composition Function 3 (N = 4)	2300
F24		Composition function 4 (N = 4)	2400
F25		Composition function 5 (N = 5)	2500
F26		Composition function 6 (N = 5)	2600
F27		Composition function 7 (N = 6)	2700
F28		Composition function 8 (N = 6)	2800
F29		Composition function 9 (N = 3)	2900
F30		Composition function 10 (N = 3)	3000

Search range: [−100, 100] Dimension: 30.

**Table 3:** Metaheuristic algorithms parameters settings.

Alg.	Parameter	Value
CHIO	$BR_{r}$	0.07
	$Max_{Age}$	100
GSA	$G_{0}$	100
	α	20
	*k*	∈ [n,2]
SAO	*A*	[2, 0]
	$f_{c}$	2
GOA	$c_{max}$	1
CMAES	$\alpha_{mu}$	2
AEFA	K(t)	Initialized at first
LCA	-	-

**Table 4:** Statistical outcomes comparing mAEFA against various other metaheuristics on CEC’17 benchmark functions.

Fun		mAEFA	AEFA	LCA	CHIO	GOA	GSA	CMA-ES	SAO
F1	min	1.51$\times \;10^{2}$	2.80 $\times \;10^{2}$	4.89 $\times \;10^{10}$	3.97 $\times \;10^{6}$	4.48 $\times \;10^{10}$	7.63 $\times \;10^{2}$	6.01 $\times \;10^{10}$	4.94 $\times \;10^{10}$
	max	1.04 $\times \;10^{4}$	5.61 $\times \;10^{3}$	1.63 $\times \;10^{11}$	2.40 $\times \;10^{8}$	9.75 $\times \;10^{10}$	3.88 $\times \;10^{8}$	1.00 $\times \;10^{11}$	7.72 $\times \;10^{10}$
	mean	2.73 $\times \;10^{3}$	2.12 $\times \;10^{3}$	9.03 $\times \;10^{10}$	9.94 $\times \;10^{7}$	6.97 $\times \;10^{10}$	4.56 $\times \;10^{7}$	8.25 $\times \;10^{10}$	6.75 $\times \;10^{10}$
	std	2.65 $\times \;10^{3}$	1.32 $\times \;10^{3}$	3.07 $\times \;10^{10}$	7.16 $\times \;10^{7}$	1.43 $\times \;10^{10}$	8.60 $\times \;10^{7}$	1.06 $\times \;10^{10}$	7.72 $\times \;10^{9}$
	rank	2	1	8	4	6	3	7	5
F3	min	7.37 $\times \;10^{4}$	7.30 $\times \;10^{4}$	1.16 $\times \;10^{5}$	9.91 $\times \;10^{4}$	1.04 $\times \;10^{5}$	7.57 $\times \;10^{4}$	1.15 $\times \;10^{5}$	8.48 $\times \;10^{4}$
	max	8.87 $\times \;10^{4}$	1.13 $\times \;10^{5}$	5.69 $\times \;10^{9}$	1.59 $\times \;10^{5}$	6.43 $\times \;10^{5}$	1.18 $\times \;10^{5}$	2.77 $\times \;10^{5}$	1.76 $\times \;10^{5}$
	mean	8.30 $\times \;10^{4}$	8.91 $\times \;10^{4}$	2.27 $\times \;10^{8}$	1.32 $\times \;10^{5}$	3.86 $\times \;10^{5}$	9.47 $\times \;10^{4}$	2.07 $\times \;10^{5}$	1.20 $\times \;10^{5}$
	std	4.25 $\times \;10^{3}$	9.26 $\times \;10^{3}$	1.03 $\times \;10^{9}$	1.81 $\times \;10^{4}$	1.10 $\times \;10^{5}$	9.40 $\times \;10^{3}$	3.18 $\times \;10^{4}$	2.37 $\times \;10^{4}$
	rank	1	2	8	5	7	3	6	4
F4	min	4.91 $\times \;10^{2}$	5.09 $\times \;10^{2}$	1.44 $\times \;10^{4}$	5.08 $\times \;10^{2}$	6.06 $\times \;10^{3}$	5.45 $\times \;10^{2}$	9.50 $\times \;10^{3}$	7.33 $\times \;10^{3}$
	max	5.86 $\times \;10^{2}$	5.81 $\times \;10^{2}$	7.34 $\times \;10^{4}$	5.93 $\times \;10^{2}$	3.28 $\times \;10^{4}$	9.26 $\times \;10^{2}$	2.97 $\times \;10^{4}$	2.56 $\times \;10^{4}$
	mean	5.27 $\times \;10^{2}$	5.34 $\times \;10^{2}$	3.60 $\times \;10^{4}$	5.50 $\times \;10^{2}$	2.09 $\times \;10^{4}$	6.54 $\times \;10^{2}$	1.98 $\times \;10^{4}$	1.85 $\times \;10^{4}$
	std	1.76 $\times \;10^{1}$	1.59 $\times \;10^{1}$	1.68 $\times \;10^{4}$	2.35 $\times \;10^{1}$	6.73 $\times \;10^{3}$	9.02 $\times \;10^{1}$	5.01 $\times \;10^{3}$	3.55 $\times \;10^{3}$
	rank	1	2	8	3	7	4	6	5
F5	min	5.17 $\times \;10^{2}$	5.52 $\times \;10^{2}$	9.37 $\times \;10^{2}$	6.26 $\times \;10^{2}$	8.98 $\times \;10^{2}$	6.80 $\times \;10^{2}$	9.82 $\times \;10^{2}$	8.88 $\times \;10^{2}$
	max	5.41 $\times \;10^{2}$	6.15 $\times \;10^{2}$	1.27 $\times \;10^{3}$	6.87 $\times \;10^{2}$	1.14 $\times \;10^{3}$	7.94 $\times \;10^{2}$	1.10 $\times \;10^{3}$	1.05 $\times \;10^{3}$
	mean	5.28 $\times \;10^{2}$	5.85 $\times \;10^{2}$	1.07 $\times \;10^{3}$	6.56 $\times \;10^{2}$	1.02 $\times \;10^{3}$	7.42 $\times \;10^{2}$	1.03 $\times \;10^{3}$	9.78 $\times \;10^{2}$
	std	6.34 $\times \;10^{0}$	1.51 $\times \;10^{1}$	9.93 $\times \;10^{1}$	1.54 $\times \;10^{1}$	6.40 $\times \;10^{1}$	2.75 $\times \;10^{1}$	3.28 $\times \;10^{1}$	3.53 $\times \;10^{1}$
	rank	1	2	8	3	6	4	7	5
F6	min	6.00 $\times \;10^{2}$	6.08 $\times \;10^{2}$	6.88 $\times \;10^{2}$	6.04 $\times \;10^{2}$	6.88 $\times \;10^{2}$	6.50 $\times \;10^{2}$	6.84 $\times \;10^{2}$	6.74 $\times \;10^{2}$
	max	6.01 $\times \;10^{2}$	6.38 $\times \;10^{2}$	7.53 $\times \;10^{2}$	6.12 $\times \;10^{2}$	7.50 $\times \;10^{2}$	6.65 $\times \;10^{2}$	7.21 $\times \;10^{2}$	7.08 $\times \;10^{2}$
	mean	6.00 $\times \;10^{2}$	6.23 $\times \;10^{2}$	7.23 $\times \;10^{2}$	6.08 $\times \;10^{2}$	7.18 $\times \;10^{2}$	6.57 $\times \;10^{2}$	7.08 $\times \;10^{2}$	6.96 $\times \;10^{2}$
	std	4.06 $\times \;10^{-1}$	7.22 $\times \;10^{0}$	2.02 $\times \;10^{1}$	1.83 $\times \;10^{0}$	1.57 $\times \;10^{1}$	3.62 $\times \;10^{0}$	1.03 $\times \;10^{1}$	7.88 $\times \;10^{0}$
	rank	1	3	8	2	7	4	6	5
F7	min	7.39 $\times \;10^{2}$	7.43 $\times \;10^{2}$	1.40 $\times \;10^{3}$	8.74 $\times \;10^{2}$	1.53 $\times \;10^{3}$	8.91 $\times \;10^{2}$	2.30 $\times \;10^{3}$	1.45 $\times \;10^{3}$
	max	7.57 $\times \;10^{2}$	7.76 $\times \;10^{2}$	3.81 $\times \;10^{3}$	9.43 $\times \;10^{2}$	3.05 $\times \;10^{3}$	1.03 $\times \;10^{3}$	3.09 $\times \;10^{3}$	1.74 $\times \;10^{3}$
	mean	7.49 $\times \;10^{2}$	7.54 $\times \;10^{2}$	2.51 $\times \;10^{3}$	9.13 $\times \;10^{2}$	1.79 $\times \;10^{3}$	9.49 $\times \;10^{2}$	2.77 $\times \;10^{3}$	1.55 $\times \;10^{3}$
	std	4.58 $\times \;10^{0}$	8.25 $\times \;10^{0}$	8.83 $\times \;10^{2}$	1.83 $\times \;10^{1}$	2.98 $\times \;10^{2}$	3.29 $\times \;10^{1}$	1.89 $\times \;10^{2}$	6.34 $\times \;10^{1}$
	rank	1	2	7	3	6	4	8	5
F8	min	8.14 $\times \;10^{2}$	8.51 $\times \;10^{2}$	1.16 $\times \;10^{3}$	9.18 $\times \;10^{2}$	1.13 $\times \;10^{3}$	9.28 $\times \;10^{2}$	1.22 $\times \;10^{3}$	1.11 $\times \;10^{3}$
	max	8.36 $\times \;10^{2}$	8.93 $\times \;10^{2}$	1.46 $\times \;10^{3}$	9.79 $\times \;10^{2}$	1.44 $\times \;10^{3}$	1.00 $\times \;10^{3}$	1.34 $\times \;10^{3}$	1.23 $\times \;10^{3}$
	mean	8.25 $\times \;10^{2}$	8.67 $\times \;10^{2}$	1.30 $\times \;10^{3}$	9.55 $\times \;10^{2}$	1.24 $\times \;10^{3}$	9.58 $\times \;10^{2}$	1.29 $\times \;10^{3}$	1.19 $\times \;10^{3}$
	std	5.63 $\times \;10^{0}$	1.20 $\times \;10^{1}$	9.98 $\times \;10^{1}$	1.63 $\times \;10^{1}$	7.13 $\times \;10^{1}$	1.82 $\times \;10^{1}$	2.69 $\times \;10^{1}$	2.79 $\times \;10^{1}$
	rank	1	2	8	3	6	4	7	5
F9	min	9.00 $\times \;10^{2}$	9.45 $\times \;10^{2}$	1.17 $\times \;10^{4}$	2.65 $\times \;10^{3}$	1.31 $\times \;10^{4}$	3.50 $\times \;10^{3}$	1.68 $\times \;10^{4}$	1.10 $\times \;10^{4}$
	max	9.77 $\times \;10^{2}$	1.60 $\times \;10^{3}$	4.70 $\times \;10^{4}$	7.51 $\times \;10^{3}$	3.35 $\times \;10^{4}$	5.21 $\times \;10^{3}$	3.05 $\times \;10^{4}$	1.90 $\times \;10^{4}$
	mean	9.15 $\times \;10^{2}$	1.24 $\times \;10^{3}$	2.75 $\times \;10^{4}$	5.18 $\times \;10^{3}$	2.13 $\times \;10^{4}$	4.24 $\times \;10^{3}$	2.49 $\times \;10^{4}$	1.43 $\times \;10^{4}$
	std	2.13 $\times \;10^{1}$	1.92 $\times \;10^{2}$	1.20 $\times \;10^{4}$	1.20 $\times \;10^{3}$	5.16 $\times \;10^{3}$	4.28 $\times \;10^{2}$	2.63 $\times \;10^{3}$	1.72 $\times \;10^{3}$
	rank	1	2	8	4	6	3	7	5
F10	min	4.45 $\times \;10^{3}$	3.87 $\times \;10^{3}$	9.35 $\times \;10^{3}$	4.30 $\times \;10^{3}$	8.08 $\times \;10^{3}$	4.25 $\times \;10^{3}$	8.19 $\times \;10^{3}$	8.44 $\times \;10^{3}$
	max	5.75 $\times \;10^{3}$	5.56 $\times \;10^{3}$	1.16 $\times \;10^{4}$	5.41 $\times \;10^{3}$	1.04 $\times \;10^{4}$	6.21 $\times \;10^{3}$	9.24 $\times \;10^{3}$	1.01 $\times \;10^{4}$
	mean	5.04 $\times \;10^{3}$	4.79 $\times \;10^{3}$	1.07 $\times \;10^{4}$	5.02 $\times \;10^{3}$	9.51 $\times \;10^{3}$	5.09 $\times \;10^{3}$	8.82 $\times \;10^{3}$	9.29 $\times \;10^{3}$
	std	2.96 $\times \;10^{2}$	4.21 $\times \;10^{2}$	5.74 $\times \;10^{2}$	2.33 $\times \;10^{2}$	5.40 $\times \;10^{2}$	5.35 $\times \;10^{2}$	2.51 $\times \;10^{2}$	4.40 $\times \;10^{2}$
	rank	3	1	8	2	7	4	5	6
F11	min	1.37 $\times \;10^{3}$	3.29 $\times \;10^{3}$	1.07 $\times \;10^{4}$	1.53 $\times \;10^{3}$	1.10 $\times \;10^{4}$	2.27 $\times \;10^{3}$	8.64 $\times \;10^{3}$	6.72 $\times \;10^{3}$
	max	5.60 $\times \;10^{3}$	9.98 $\times \;10^{3}$	2.61 $\times \;10^{5}$	4.80 $\times \;10^{3}$	5.75 $\times \;10^{4}$	7.76 $\times \;10^{3}$	2.04 $\times \;10^{4}$	1.56 $\times \;10^{4}$
	mean	2.79 $\times \;10^{3}$	6.87 $\times \;10^{3}$	4.19 $\times \;10^{4}$	2.38 $\times \;10^{3}$	3.29 $\times \;10^{4}$	4.42 $\times \;10^{3}$	1.44 $\times \;10^{4}$	1.17 $\times \;10^{4}$
	std	1.04 $\times \;10^{3}$	1.67 $\times \;10^{3}$	5.08 $\times \;10^{4}$	6.88 $\times \;10^{2}$	1.21 $\times \;10^{4}$	1.15 $\times \;10^{3}$	3.30 $\times \;10^{3}$	2.14 $\times \;10^{3}$
	rank	2	4	8	1	7	3	6	5
F12	min	3.91 $\times \;10^{5}$	2.25 $\times \;10^{6}$	7.45 $\times \;10^{9}$	4.62 $\times \;10^{6}$	4.76 $\times \;10^{9}$	2.95 $\times \;10^{6}$	6.18 $\times \;10^{9}$	8.81 $\times \;10^{9}$
	max	1.13 $\times \;10^{7}$	2.60 $\times \;10^{7}$	3.66 $\times \;10^{10}$	2.69 $\times \;10^{7}$	2.12 $\times \;10^{10}$	2.51 $\times \;10^{8}$	1.68 $\times \;10^{10}$	2.27 $\times \;10^{10}$
	mean	3.47 $\times \;10^{6}$	1.05 $\times \;10^{7}$	2.24 $\times \;10^{10}$	1.25 $\times \;10^{7}$	1.15 $\times \;10^{10}$	8.39 $\times \;10^{7}$	1.10 $\times \;10^{10}$	1.70 $\times \;10^{10}$
	std	2.70 $\times \;10^{6}$	6.10 $\times \;10^{6}$	7.68 $\times \;10^{9}$	5.32 $\times \;10^{6}$	4.05 $\times \;10^{9}$	7.17 $\times \;10^{7}$	2.19 $\times \;10^{9}$	3.23 $\times \;10^{9}$
	rank	1	2	8	3	6	4	5	7
F13	min	4.04 $\times \;10^{3}$	1.48 $\times \;10^{4}$	5.99 $\times \;10^{9}$	9.32 $\times \;10^{5}$	2.51 $\times \;10^{9}$	2.22 $\times \;10^{4}$	2.97 $\times \;10^{9}$	2.61 $\times \;10^{9}$
	max	6.38 $\times \;10^{4}$	6.22 $\times \;10^{4}$	4.36 $\times \;10^{10}$	1.69 $\times \;10^{7}$	2.00 $\times \;10^{10}$	4.84 $\times \;10^{4}$	9.81 $\times \;10^{9}$	1.82 $\times \;10^{10}$
	mean	2.91 $\times \;10^{4}$	3.30 $\times \;10^{4}$	2.04 $\times \;10^{10}$	6.41 $\times \;10^{6}$	7.77 $\times \;10^{9}$	3.25 $\times \;10^{4}$	5.85 $\times \;10^{9}$	9.74 $\times \;10^{9}$
	std	1.61 $\times \;10^{4}$	1.16 $\times \;10^{4}$	1.06 $\times \;10^{10}$	3.73 $\times \;10^{6}$	3.67 $\times \;10^{9}$	6.89 $\times \;10^{3}$	1.88 $\times \;10^{9}$	4.29 $\times \;10^{9}$
	rank	1	3	8	4	6	2	5	7
F14	min	2.50 $\times \;10^{5}$	3.72 $\times \;10^{5}$	3.06 $\times \;10^{6}$	1.52 $\times \;10^{4}$	9.88 $\times \;10^{5}$	5.58 $\times \;10^{5}$	4.97 $\times \;10^{5}$	1.55 $\times \;10^{5}$
	max	2.38 $\times \;10^{6}$	2.17 $\times \;10^{6}$	2.33 $\times \;10^{8}$	1.19 $\times \;10^{6}$	3.21 $\times \;10^{7}$	1.91 $\times \;10^{6}$	4.51 $\times \;10^{6}$	4.25 $\times \;10^{7}$
	mean	1.22 $\times \;10^{6}$	1.23 $\times \;10^{6}$	5.39 $\times \;10^{7}$	4.23 $\times \;10^{5}$	7.73 $\times \;10^{6}$	1.11 $\times \;10^{6}$	1.87 $\times \;10^{6}$	6.26 $\times \;10^{6}$
	std	5.41 $\times \;10^{5}$	4.59 $\times \;10^{5}$	5.83 $\times \;10^{7}$	2.83 $\times \;10^{5}$	7.57 $\times \;10^{6}$	3.79 $\times \;10^{5}$	1.26 $\times \;10^{6}$	8.83 $\times \;10^{6}$
	rank	3	4	8	1	7	2	5	6
F15	min	2.20 $\times \;10^{3}$	8.99 $\times \;10^{3}$	7.26 $\times \;10^{8}$	5.15 $\times \;10^{4}$	1.94 $\times \;10^{8}$	9.06 $\times \;10^{3}$	2.11 $\times \;10^{8}$	3.51 $\times \;10^{7}$
	max	1.73 $\times \;10^{4}$	3.53 $\times \;10^{4}$	1.19 $\times \;10^{10}$	3.42 $\times \;10^{6}$	4.82 $\times \;10^{9}$	2.47 $\times \;10^{4}$	1.10 $\times \;10^{9}$	3.89 $\times \;10^{9}$
	mean	6.74 $\times \;10^{3}$	1.61 $\times \;10^{4}$	5.16 $\times \;10^{9}$	8.77 $\times \;10^{5}$	1.60 $\times \;10^{9}$	1.53 $\times \;10^{4}$	6.69 $\times \;10^{8}$	1.62 $\times \;10^{9}$
	std	4.02 $\times \;10^{3}$	5.63 $\times \;10^{3}$	3.24 $\times \;10^{9}$	7.52 $\times \;10^{5}$	1.14 $\times \;10^{9}$	3.41 $\times \;10^{3}$	2.02 $\times \;10^{8}$	1.09 $\times \;10^{9}$
	rank	1	3	8	4	6	2	5	7
F16	min	1.99 $\times \;10^{3}$	2.48 $\times \;10^{3}$	6.00 $\times \;10^{3}$	2.15 $\times \;10^{3}$	4.52 $\times \;10^{3}$	2.86 $\times \;10^{3}$	4.84 $\times \;10^{3}$	4.91 $\times \;10^{3}$
	max	3.03 $\times \;10^{3}$	3.55 $\times \;10^{3}$	1.68 $\times \;10^{4}$	2.97 $\times \;10^{3}$	7.17 $\times \;10^{3}$	4.16 $\times \;10^{3}$	5.75 $\times \;10^{3}$	9.79 $\times \;10^{3}$
	mean	2.53 $\times \;10^{3}$	2.93 $\times \;10^{3}$	8.98 $\times \;10^{3}$	2.64 $\times \;10^{3}$	5.15 $\times \;10^{3}$	3.51 $\times \;10^{3}$	5.33 $\times \;10^{3}$	7.03 $\times \;10^{3}$
	std	3.02 $\times \;10^{2}$	2.71 $\times \;10^{2}$	2.23 $\times \;10^{3}$	1.81 $\times \;10^{2}$	6.19 $\times \;10^{2}$	3.34 $\times \;10^{2}$	2.90 $\times \;10^{2}$	1.42 $\times \;10^{3}$
	rank	1	3	8	2	5	4	6	7
F17	min	1.79 $\times \;10^{3}$	2.15 $\times \;10^{3}$	3.40 $\times \;10^{3}$	1.90 $\times \;10^{3}$	3.24 $\times \;10^{3}$	2.50 $\times \;10^{3}$	3.16 $\times \;10^{3}$	3.18 $\times \;10^{3}$
	max	2.59 $\times \;10^{3}$	3.40 $\times \;10^{3}$	3.39 $\times \;10^{5}$	2.27 $\times \;10^{3}$	7.46 $\times \;10^{3}$	3.38 $\times \;10^{3}$	4.18 $\times \;10^{3}$	2.40 $\times \;10^{4}$
	mean	2.21 $\times \;10^{3}$	2.61 $\times \;10^{3}$	3.85 $\times \;10^{4}$	2.11 $\times \;10^{3}$	4.10 $\times \;10^{3}$	2.93 $\times \;10^{3}$	3.63 $\times \;10^{3}$	6.71 $\times \;10^{3}$
	std	2.02 $\times \;10^{2}$	2.28 $\times \;10^{2}$	7.27 $\times \;10^{4}$	1.07 $\times \;10^{2}$	8.46 $\times \;10^{2}$	2.37 $\times \;10^{2}$	2.67 $\times \;10^{2}$	4.57 $\times \;10^{3}$
	rank	2	3	8	1	6	4	5	7
F18	min	5.48 $\times \;10^{4}$	1.40 $\times \;10^{5}$	5.68 $\times \;10^{7}$	2.47 $\times \;10^{5}$	6.98 $\times \;10^{6}$	9.68 $\times \;10^{4}$	6.99 $\times \;10^{6}$	5.36 $\times \;10^{6}$
	max	3.48 $\times \;10^{6}$	5.55 $\times \;10^{6}$	1.96 $\times \;10^{9}$	2.93 $\times \;10^{6}$	2.46 $\times \;10^{8}$	8.86 $\times \;10^{5}$	6.05 $\times \;10^{7}$	3.68 $\times \;10^{8}$
	mean	7.18 $\times \;10^{5}$	1.76 $\times \;10^{6}$	5.90 $\times \;10^{8}$	9.86 $\times \;10^{5}$	6.51 $\times \;10^{7}$	4.39 $\times \;10^{5}$	3.22 $\times \;10^{7}$	1.06 $\times \;10^{8}$
	std	7.67 $\times \;10^{5}$	1.43 $\times \;10^{6}$	4.61 $\times \;10^{8}$	6.19 $\times \;10^{5}$	5.47 $\times \;10^{7}$	2.41 $\times \;10^{5}$	1.25 $\times \;10^{7}$	9.89 $\times \;10^{7}$
	rank	2	4	8	3	6	1	5	7
F19	min	2.20 $\times \;10^{3}$	4.93 $\times \;10^{4}$	3.51 $\times \;10^{8}$	3.26 $\times \;10^{4}$	3.66 $\times \;10^{8}$	5.79 $\times \;10^{4}$	2.75 $\times \;10^{8}$	4.20 $\times \;10^{7}$
	max	1.48 $\times \;10^{4}$	4.14 $\times \;10^{6}$	1.62 $\times \;10^{10}$	1.32 $\times \;10^{6}$	7.46 $\times \;10^{9}$	4.73 $\times \;10^{5}$	1.73 $\times \;10^{9}$	2.90 $\times \;10^{9}$
	mean	6.60 $\times \;10^{3}$	9.02 $\times \;10^{5}$	6.00 $\times \;10^{9}$	5.97 $\times \;10^{5}$	2.06 $\times \;10^{9}$	1.99 $\times \;10^{5}$	9.98 $\times \;10^{8}$	1.24 $\times \;10^{9}$
	std	3.01 $\times \;10^{3}$	8.85 $\times \;10^{5}$	4.41 $\times \;10^{9}$	4.22 $\times \;10^{5}$	1.50 $\times \;10^{9}$	9.72 $\times \;10^{4}$	3.79 $\times \;10^{8}$	7.84 $\times \;10^{8}$
	rank	1	4	8	3	7	2	5	6
F20	min	2.65 $\times \;10^{3}$	2.30 $\times \;10^{3}$	3.24 $\times \;10^{3}$	2.23 $\times \;10^{3}$	2.89 $\times \;10^{3}$	2.75 $\times \;10^{3}$	2.76 $\times \;10^{3}$	2.84 $\times \;10^{3}$
	max	3.10 $\times \;10^{3}$	3.36 $\times \;10^{3}$	4.24 $\times \;10^{3}$	2.64 $\times \;10^{3}$	3.74 $\times \;10^{3}$	3.44 $\times \;10^{3}$	3.24 $\times \;10^{3}$	3.48 $\times \;10^{3}$
	mean	2.87 $\times \;10^{3}$	2.83 $\times \;10^{3}$	3.72 $\times \;10^{3}$	2.41 $\times \;10^{3}$	3.41 $\times \;10^{3}$	3.08 $\times \;10^{3}$	3.05 $\times \;10^{3}$	3.19 $\times \;10^{3}$
	std	1.41 $\times \;10^{2}$	2.49 $\times \;10^{2}$	2.55 $\times \;10^{2}$	9.71 $\times \;10^{1}$	1.67 $\times \;10^{2}$	1.76 $\times \;10^{2}$	1.08 $\times \;10^{2}$	1.62 $\times \;10^{2}$
	rank	3	2	8	1	7	5	4	6
F21	min	2.31 $\times \;10^{3}$	2.36 $\times \;10^{3}$	2.76 $\times \;10^{3}$	2.24 $\times \;10^{3}$	2.64 $\times \;10^{3}$	2.58 $\times \;10^{3}$	2.69 $\times \;10^{3}$	2.69 $\times \;10^{3}$
	max	2.35 $\times \;10^{3}$	2.46 $\times \;10^{3}$	3.06 $\times \;10^{3}$	2.49 $\times \;10^{3}$	2.88 $\times \;10^{3}$	2.71 $\times \;10^{3}$	2.83 $\times \;10^{3}$	2.90 $\times \;10^{3}$
	mean	2.33 $\times \;10^{3}$	2.40 $\times \;10^{3}$	2.90 $\times \;10^{3}$	2.44 $\times \;10^{3}$	2.78 $\times \;10^{3}$	2.64 $\times \;10^{3}$	2.77 $\times \;10^{3}$	2.81 $\times \;10^{3}$
	std	8.00 $\times \;10^{0}$	1.89 $\times \;10^{1}$	8.88 $\times \;10^{1}$	7.77 $\times \;10^{1}$	5.60 $\times \;10^{1}$	3.95 $\times \;10^{1}$	3.70 $\times \;10^{1}$	5.31 $\times \;10^{1}$
	rank	1	2	8	3	6	4	5	7
F22	min	2.30 $\times \;10^{3}$	2.30 $\times \;10^{3}$	8.68 $\times \;10^{3}$	2.43 $\times \;10^{3}$	9.10 $\times \;10^{3}$	6.37 $\times \;10^{3}$	9.67 $\times \;10^{3}$	9.37 $\times \;10^{3}$
	max	2.30 $\times \;10^{3}$	2.32 $\times \;10^{3}$	1.33 $\times \;10^{4}$	6.85 $\times \;10^{3}$	1.16 $\times \;10^{4}$	8.30 $\times \;10^{3}$	1.06 $\times \;10^{4}$	1.12 $\times \;10^{4}$
	mean	2.30 $\times \;10^{3}$	2.30 $\times \;10^{3}$	1.19 $\times \;10^{4}$	2.95 $\times \;10^{3}$	1.07 $\times \;10^{4}$	7.31 $\times \;10^{3}$	1.01 $\times \;10^{4}$	1.04 $\times \;10^{4}$
	std	7.55 $\times \;10^{-1}$	4.21 $\times \;10^{0}$	1.19 $\times \;10^{3}$	1.22 $\times \;10^{3}$	5.89 $\times \;10^{2}$	4.72 $\times \;10^{2}$	2.15 $\times \;10^{2}$	4.09 $\times \;10^{2}$
	rank	1	2	8	3	7	4	5	6
F23	min	2.67 $\times \;10^{3}$	2.77 $\times \;10^{3}$	3.49 $\times \;10^{3}$	2.70 $\times \;10^{3}$	3.08 $\times \;10^{3}$	3.47 $\times \;10^{3}$	3.13 $\times \;10^{3}$	3.44 $\times \;10^{3}$
	max	2.72 $\times \;10^{3}$	3.00 $\times \;10^{3}$	4.35 $\times \;10^{3}$	2.85 $\times \;10^{3}$	3.50 $\times \;10^{3}$	4.18 $\times \;10^{3}$	3.40 $\times \;10^{3}$	4.19 $\times \;10^{3}$
	mean	2.69 $\times \;10^{3}$	2.87 $\times \;10^{3}$	3.84 $\times \;10^{3}$	2.79 $\times \;10^{3}$	3.26 $\times \;10^{3}$	3.83 $\times \;10^{3}$	3.31 $\times \;10^{3}$	3.73 $\times \;10^{3}$
	std	1.14 $\times \;10^{1}$	4.51 $\times \;10^{1}$	2.26 $\times \;10^{2}$	4.49 $\times \;10^{1}$	9.72 $\times \;10^{1}$	1.63 $\times \;10^{2}$	5.06 $\times \;10^{1}$	1.65 $\times \;10^{2}$
	rank	1	3	8	2	4	7	5	6
F24	min	2.84 $\times \;10^{3}$	2.87 $\times \;10^{3}$	3.81 $\times \;10^{3}$	2.98 $\times \;10^{3}$	3.13 $\times \;10^{3}$	3.37 $\times \;10^{3}$	3.34 $\times \;10^{3}$	3.50 $\times \;10^{3}$
	max	2.88 $\times \;10^{3}$	3.00 $\times \;10^{3}$	4.88 $\times \;10^{3}$	3.11 $\times \;10^{3}$	3.70 $\times \;10^{3}$	3.67 $\times \;10^{3}$	3.54 $\times \;10^{3}$	4.08 $\times \;10^{3}$
	mean	2.86 $\times \;10^{3}$	2.94 $\times \;10^{3}$	4.26 $\times \;10^{3}$	3.05 $\times \;10^{3}$	3.35 $\times \;10^{3}$	3.50 $\times \;10^{3}$	3.45 $\times \;10^{3}$	3.81 $\times \;10^{3}$
	std	1.17 $\times \;10^{1}$	3.02 $\times \;10^{1}$	2.70 $\times \;10^{2}$	3.34 $\times \;10^{1}$	1.46 $\times \;10^{2}$	6.55 $\times \;10^{1}$	5.72 $\times \;10^{1}$	1.48 $\times \;10^{2}$
	rank	1	2	8	3	4	6	5	7
F25	min	2.90 $\times \;10^{3}$	2.93 $\times \;10^{3}$	4.92 $\times \;10^{3}$	2.91 $\times \;10^{3}$	5.12 $\times \;10^{3}$	2.93 $\times \;10^{3}$	7.37 $\times \;10^{3}$	4.72 $\times \;10^{3}$
	max	2.97 $\times \;10^{3}$	3.02 $\times \;10^{3}$	2.40 $\times \;10^{4}$	2.97 $\times \;10^{3}$	1.41 $\times \;10^{4}$	3.05 $\times \;10^{3}$	1.41 $\times \;10^{4}$	6.94 $\times \;10^{3}$
	mean	2.93 $\times \;10^{3}$	2.98 $\times \;10^{3}$	1.23 $\times \;10^{4}$	2.94 $\times \;10^{3}$	8.03 $\times \;10^{3}$	2.99 $\times \;10^{3}$	1.08 $\times \;10^{4}$	5.79 $\times \;10^{3}$
	std	2.14 $\times \;10^{1}$	2.40 $\times \;10^{1}$	6.06 $\times \;10^{3}$	1.66 $\times \;10^{1}$	2.14 $\times \;10^{3}$	2.01 $\times \;10^{1}$	1.53 $\times \;10^{3}$	5.14 $\times \;10^{2}$
	rank	1	3	8	2	6	4	7	5
F26	min	2.93 $\times \;10^{3}$	2.80 $\times \;10^{3}$	1.09 $\times \;10^{4}$	3.27 $\times \;10^{3}$	8.78 $\times \;10^{3}$	6.32 $\times \;10^{3}$	1.04 $\times \;10^{4}$	1.08 $\times \;10^{4}$
	max	6.16 $\times \;10^{3}$	7.00 $\times \;10^{3}$	2.06 $\times \;10^{4}$	5.43 $\times \;10^{3}$	1.39 $\times \;10^{4}$	9.01 $\times \;10^{3}$	1.21 $\times \;10^{4}$	1.45 $\times \;10^{4}$
	mean	4.36 $\times \;10^{3}$	4.50 $\times \;10^{3}$	1.47 $\times \;10^{4}$	3.93 $\times \;10^{3}$	1.11 $\times \;10^{4}$	7.79 $\times \;10^{3}$	1.11 $\times \;10^{4}$	1.32 $\times \;10^{4}$
	std	6.60 $\times \;10^{2}$	1.34 $\times \;10^{3}$	2.84 $\times \;10^{3}$	5.49 $\times \;10^{2}$	1.29 $\times \;10^{3}$	5.70 $\times \;10^{2}$	4.56 $\times \;10^{2}$	8.17 $\times \;10^{2}$
	rank	2	3	8	1	5	4	6	7
F27	min	3.22 $\times \;10^{3}$	3.31 $\times \;10^{3}$	4.18 $\times \;10^{3}$	3.22 $\times \;10^{3}$	3.20 $\times \;10^{3}$	4.43 $\times \;10^{3}$	3.42 $\times \;10^{3}$	3.86 $\times \;10^{3}$
	max	3.27 $\times \;10^{3}$	3.55 $\times \;10^{3}$	6.35 $\times \;10^{3}$	3.25 $\times \;10^{3}$	3.80 $\times \;10^{3}$	5.65 $\times \;10^{3}$	3.80 $\times \;10^{3}$	5.80 $\times \;10^{3}$
	mean	3.24 $\times \;10^{3}$	3.41 $\times \;10^{3}$	5.31 $\times \;10^{3}$	3.23 $\times \;10^{3}$	3.22 $\times \;10^{3}$	5.03 $\times \;10^{3}$	3.66 $\times \;10^{3}$	4.80 $\times \;10^{3}$
	std	1.46 $\times \;10^{1}$	5.33 $\times \;10^{1}$	5.13 $\times \;10^{2}$	6.12 $\times \;10^{0}$	1.10 $\times \;10^{2}$	3.23 $\times \;10^{2}$	9.67 $\times \;10^{1}$	5.53 $\times \;10^{2}$
	rank	3	4	8	2	1	7	5	6
F28	min	3.24 $\times \;10^{3}$	3.30 $\times \;10^{3}$	6.87 $\times \;10^{3}$	3.30 $\times \;10^{3}$	3.30 $\times \;10^{3}$	3.40 $\times \;10^{3}$	6.11 $\times \;10^{3}$	3.31 $\times \;10^{3}$
	max	3.37 $\times \;10^{3}$	3.41 $\times \;10^{3}$	1.52 $\times \;10^{4}$	3.39 $\times \;10^{3}$	3.30 $\times \;10^{3}$	4.11 $\times \;10^{3}$	9.24 $\times \;10^{3}$	9.42 $\times \;10^{3}$
	mean	3.31 $\times \;10^{3}$	3.35 $\times \;10^{3}$	1.13 $\times \;10^{4}$	3.34 $\times \;10^{3}$	3.30 $\times \;10^{3}$	3.59 $\times \;10^{3}$	8.12 $\times \;10^{3}$	6.46 $\times \;10^{3}$
	std	2.80 $\times \;10^{1}$	2.90 $\times \;10^{1}$	2.37 $\times \;10^{3}$	1.94 $\times \;10^{1}$	2.33 $\times \;10^{-3}$	1.87 $\times \;10^{2}$	8.01 $\times \;10^{2}$	2.28 $\times \;10^{3}$
	rank	2	4	8	3	1	5	7	6
F29	min	3.50 $\times \;10^{3}$	4.46 $\times \;10^{3}$	6.11 $\times \;10^{3}$	3.52 $\times \;10^{3}$	5.18 $\times \;10^{3}$	4.94 $\times \;10^{3}$	5.13 $\times \;10^{3}$	5.98 $\times \;10^{3}$
	max	4.41 $\times \;10^{3}$	5.53 $\times \;10^{3}$	1.55 $\times \;10^{5}$	3.99 $\times \;10^{3}$	1.13 $\times \;10^{4}$	6.16 $\times \;10^{3}$	6.97 $\times \;10^{3}$	1.87 $\times \;10^{4}$
	mean	3.94 $\times \;10^{3}$	4.86 $\times \;10^{3}$	3.74 $\times \;10^{4}$	3.77 $\times \;10^{3}$	7.29 $\times \;10^{3}$	5.42 $\times \;10^{3}$	6.19 $\times \;10^{3}$	9.73 $\times \;10^{3}$
	std	2.28 $\times \;10^{2}$	2.41 $\times \;10^{2}$	3.90 $\times \;10^{4}$	1.02 $\times \;10^{2}$	1.56 $\times \;10^{3}$	2.88 $\times \;10^{2}$	4.29 $\times \;10^{2}$	3.34 $\times \;10^{3}$
	rank	2	3	8	1	6	4	5	7
F30	min	2.60 $\times \;10^{4}$	2.44 $\times \;10^{6}$	1.35 $\times \;10^{9}$	1.29 $\times \;10^{5}$	5.02 $\times \;10^{8}$	1.35 $\times \;10^{6}$	2.74 $\times \;10^{8}$	4.59 $\times \;10^{8}$
	max	1.49 $\times \;10^{6}$	1.18 $\times \;10^{7}$	8.48 $\times \;10^{9}$	2.80 $\times \;10^{6}$	3.74 $\times \;10^{9}$	5.28 $\times \;10^{6}$	1.14 $\times \;10^{9}$	7.19 $\times \;10^{9}$
	mean	3.23 $\times \;10^{5}$	5.72 $\times \;10^{6}$	3.98 $\times \;10^{9}$	6.71 $\times \;10^{5}$	1.35 $\times \;10^{9}$	3.17 $\times \;10^{6}$	6.92 $\times \;10^{8}$	2.38 $\times \;10^{9}$
	std	3.59 $\times \;10^{5}$	2.22 $\times \;10^{6}$	1.83 $\times \;10^{9}$	5.39 $\times \;10^{5}$	7.31 $\times \;10^{8}$	9.97 $\times \;10^{5}$	2.31 $\times \;10^{8}$	1.61 $\times \;10^{9}$
	rank	1	4	8	2	6	3	5	7

**Table 5:** Wilcoxon rank-sum test results for the comparative algorithms against the proposed mAEFA using CEC’17 benchmark functions, where a = 0.05 and Dim = 30.

mAEFA Vs	AEFA	LCA	CHIO	GOA	GSA	CMA-ES	SAO
F1	9.59 $\times \;10^{-01}$	3.02 $\times \;10^{-11}$	3.02 $\times \;10^{-11}$	3.02 $\times \;10^{-11}$	3.18 $\times \;10^{-04}$	3.02 $\times \;10^{-11}$	3.02 $\times \;10^{-11}$
F3	1.52 $\times \;10^{-03}$	3.02 $\times \;10^{-11}$	3.02 $\times \;10^{-11}$	3.02 $\times \;10^{-11}$	2.78 $\times \;10^{-07}$	3.02 $\times \;10^{-11}$	9.92 $\times \;10^{-11}$
F4	2.92 $\times \;10^{-02}$	3.02 $\times \;10^{-11}$	1.17 $\times \;10^{-04}$	3.02 $\times \;10^{-11}$	6.70 $\times \;10^{-11}$	3.02 $\times \;10^{-11}$	3.02 $\times \;10^{-11}$
F5	3.00 $\times \;10^{-11}$	3.00 $\times \;10^{-11}$	3.00 $\times \;10^{-11}$	3.00 $\times \;10^{-11}$	3.00 $\times \;10^{-11}$	3.00 $\times \;10^{-11}$	3.00 $\times \;10^{-11}$
F6	3.02 $\times \;10^{-11}$	3.02 $\times \;10^{-11}$	3.02 $\times \;10^{-11}$	3.02 $\times \;10^{-11}$	3.02 $\times \;10^{-11}$	3.02 $\times \;10^{-11}$	3.02 $\times \;10^{-11}$
F7	1.19 $\times \;10^{-01}$	3.02 $\times \;10^{-11}$	3.02 $\times \;10^{-11}$	3.02 $\times \;10^{-11}$	3.02 $\times \;10^{-11}$	3.02 $\times \;10^{-11}$	3.02 $\times \;10^{-11}$
F8	3.00 $\times \;10^{-11}$	3.01 $\times \;10^{-11}$	3.01 $\times \;10^{-11}$	3.01 $\times \;10^{-11}$	3.01 $\times \;10^{-11}$	3.01 $\times \;10^{-11}$	3.01 $\times \;10^{-11}$
F9	4.50 $\times \;10^{-11}$	3.02 $\times \;10^{-11}$	3.02 $\times \;10^{-11}$	3.02 $\times \;10^{-11}$	3.02 $\times \;10^{-11}$	3.02 $\times \;10^{-11}$	3.02 $\times \;10^{-11}$
F10	2.81 $\times \;10^{-02}$	3.02 $\times \;10^{-11}$	4.92 $\times \;10^{-01}$	3.02 $\times \;10^{-11}$	7.96 $\times \;10^{-01}$	3.02 $\times \;10^{-11}$	3.02 $\times \;10^{-11}$
F11	1.46 $\times \;10^{-10}$	3.02 $\times \;10^{-11}$	1.22 $\times \;10^{-01}$	3.02 $\times \;10^{-11}$	1.29 $\times \;10^{-06}$	3.02 $\times \;10^{-11}$	3.02 $\times \;10^{-11}$
F12	3.01 $\times \;10^{-07}$	3.02 $\times \;10^{-11}$	2.03 $\times \;10^{-09}$	3.02 $\times \;10^{-11}$	8.89 $\times \;10^{-10}$	3.02 $\times \;10^{-11}$	3.02 $\times \;10^{-11}$
F13	1.58 $\times \;10^{-01}$	3.02 $\times \;10^{-11}$	3.02 $\times \;10^{-11}$	3.02 $\times \;10^{-11}$	9.63 $\times \;10^{-02}$	3.02 $\times \;10^{-11}$	3.02 $\times \;10^{-11}$
F14	8.88 $\times \;10^{-01}$	3.02 $\times \;10^{-11}$	4.31 $\times \;10^{-08}$	4.69 $\times \;10^{-08}$	5.01 $\times \;10^{-01}$	1.26 $\times \;10^{-01}$	6.36 $\times \;10^{-05}$
F15	7.12 $\times \;10^{-09}$	3.02 $\times \;10^{-11}$	3.02 $\times \;10^{-11}$	3.02 $\times \;10^{-11}$	1.10 $\times \;10^{-08}$	3.02 $\times \;10^{-11}$	3.02 $\times \;10^{-11}$
F16	2.13 $\times \;10^{-05}$	3.02 $\times \;10^{-11}$	6.79 $\times \;10^{-02}$	3.02 $\times \;10^{-11}$	8.99 $\times \;10^{-11}$	3.02 $\times \;10^{-11}$	3.02 $\times \;10^{-11}$
F17	1.56 $\times \;10^{-08}$	3.02 $\times \;10^{-11}$	3.03 $\times \;10^{-02}$	3.02 $\times \;10^{-11}$	5.49 $\times \;10^{-11}$	3.02 $\times \;10^{-11}$	3.02 $\times \;10^{-11}$
F18	4.22 $\times \;10^{-04}$	3.02 $\times \;10^{-11}$	1.50 $\times \;10^{-02}$	3.02 $\times \;10^{-11}$	5.40 $\times \;10^{-01}$	3.02 $\times \;10^{-11}$	3.02 $\times \;10^{-11}$
F19	3.02 $\times \;10^{-11}$	3.02 $\times \;10^{-11}$	3.02 $\times \;10^{-11}$	3.02 $\times \;10^{-11}$	3.02 $\times \;10^{-11}$	3.02 $\times \;10^{-11}$	3.02 $\times \;10^{-11}$
F20	2.58 $\times \;10^{-01}$	3.02 $\times \;10^{-11}$	3.02 $\times \;10^{-11}$	8.15 $\times \;10^{-11}$	1.53 $\times \;10^{-05}$	6.74 $\times \;10^{-06}$	2.39 $\times \;10^{-08}$
F21	3.02 $\times \;10^{-11}$	3.02 $\times \;10^{-11}$	8.88 $\times \;10^{-06}$	3.02 $\times \;10^{-11}$	3.02 $\times \;10^{-11}$	3.02 $\times \;10^{-11}$	3.02 $\times \;10^{-11}$
F22	6.59 $\times \;10^{-06}$	2.88 $\times \;10^{-11}$	2.88 $\times \;10^{-11}$	2.88 $\times \;10^{-11}$	2.88 $\times \;10^{-11}$	2.88 $\times \;10^{-11}$	2.88 $\times \;10^{-11}$
F23	3.02 $\times \;10^{-11}$	3.02 $\times \;10^{-11}$	1.09 $\times \;10^{-10}$	3.02 $\times \;10^{-11}$	3.02 $\times \;10^{-11}$	3.02 $\times \;10^{-11}$	3.02 $\times \;10^{-11}$
F24	5.49 $\times \;10^{-11}$	3.02 $\times \;10^{-11}$	3.02 $\times \;10^{-11}$	3.02 $\times \;10^{-11}$	3.02 $\times \;10^{-11}$	3.02 $\times \;10^{-11}$	3.02 $\times \;10^{-11}$
F25	1.01 $\times \;10^{-08}$	3.02 $\times \;10^{-11}$	8.24 $\times \;10^{-02}$	3.02 $\times \;10^{-11}$	2.15 $\times \;10^{-10}$	3.02 $\times \;10^{-11}$	3.02 $\times \;10^{-11}$
F26	7.96 $\times \;10^{-01}$	3.02 $\times \;10^{-11}$	1.41 $\times \;10^{-04}$	3.02 $\times \;10^{-11}$	3.02 $\times \;10^{-11}$	3.02 $\times \;10^{-11}$	3.02 $\times \;10^{-11}$
F27	3.02 $\times \;10^{-11}$	3.02 $\times \;10^{-11}$	2.68 $\times \;10^{-06}$	5.57 $\times \;10^{-10}$	3.02 $\times \;10^{-11}$	3.02 $\times \;10^{-11}$	3.02 $\times \;10^{-11}$
F28	2.57 $\times \;10^{-07}$	3.02 $\times \;10^{-11}$	3.59 $\times \;10^{-05}$	2.71 $\times \;10^{-02}$	3.02 $\times \;10^{-11}$	3.02 $\times \;10^{-11}$	1.55 $\times \;10^{-09}$
F29	3.02 $\times \;10^{-11}$	3.02 $\times \;10^{-11}$	7.30 $\times \;10^{-04}$	3.02 $\times \;10^{-11}$	3.02 $\times \;10^{-11}$	3.02 $\times \;10^{-11}$	3.02 $\times \;10^{-11}$
F30	3.02 $\times \;10^{-11}$	3.02 $\times \;10^{-11}$	4.71 $\times \;10^{-04}$	3.02 $\times \;10^{-11}$	3.34 $\times \;10^{-11}$	3.02 $\times \;10^{-11}$	3.02 $\times \;10^{-11}$

**Table 6:** Statistical outcomes comparing mAEFA against FDB variants on CEC’17 benchmark functions.

Fun.		mAEFA	dfDB-MRFO	FDB-SOS	Fun.		mAEFA	dfDB-MRFO	FDB-SOS
F1	min	1.04 $\times \;10^{2}$	1.01 $\times \;10^{2}$	1.01 $\times \;10^{2}$	F3	min	7.63 $\times \;10^{4}$	1.46 $\times \;10^{4}$	5.50 $\times \;10^{3}$
	max	1.82 $\times \;10^{4}$	1.04 $\times \;10^{4}$	1.80 $\times \;10^{4}$		max	8.84 $\times \;10^{4}$	4.51 $\times \;10^{4}$	2.09 $\times \;10^{4}$
	avg	3.30 $\times \;10^{3}$	2.58 $\times \;10^{3}$	4.05 $\times \;10^{3}$		avg	8.40 $\times \;10^{4}$	2.83 $\times \;10^{4}$	1.20 $\times \;10^{4}$
	STD	4.12 $\times \;10^{3}$	2.70 $\times \;10^{3}$	4.51 $\times \;10^{3}$		STD	3.00 $\times \;10^{3}$	7.58 $\times \;10^{3}$	3.66 $\times \;10^{3}$
	rank	2	1	3		rank	3	2	1
F4	min	4.94 $\times \;10^{2}$	4.21 $\times \;10^{2}$	4.04 $\times \;10^{2}$	F5	min	5.17 $\times \;10^{2}$	6.52 $\times \;10^{2}$	5.73 $\times \;10^{2}$
	max	5.74 $\times \;10^{2}$	5.25 $\times \;10^{2}$	5.30 $\times \;10^{2}$		max	5.41 $\times \;10^{2}$	7.38 $\times \;10^{2}$	7.63 $\times \;10^{2}$
	avg	5.25 $\times \;10^{2}$	4.84 $\times \;10^{2}$	4.81 $\times \;10^{2}$		avg	5.25 $\times \;10^{2}$	6.96 $\times \;10^{2}$	6.36 $\times \;10^{2}$
	STD	1.26 $\times \;10^{1}$	2.69 $\times \;10^{1}$	2.71 $\times \;10^{1}$		STD	5.71 $\times \;10^{0}$	1.86 $\times \;10^{1}$	4.75 $\times \;10^{1}$
	rank	3	2	1		rank	1	3	2
F6	min	6.00 $\times \;10^{2}$	6.00 $\times \;10^{2}$	6.00 $\times \;10^{2}$	F7	min	7.41 $\times \;10^{2}$	9.24 $\times \;10^{2}$	7.98 $\times \;10^{2}$
	max	6.02 $\times \;10^{2}$	6.03 $\times \;10^{2}$	6.28 $\times \;10^{2}$		max	7.64 $\times \;10^{2}$	9.82 $\times \;10^{2}$	1.02 $\times \;10^{3}$
	avg	6.00 $\times \;10^{2}$	6.01 $\times \;10^{2}$	6.04 $\times \;10^{2}$		avg	7.49 $\times \;10^{2}$	9.52 $\times \;10^{2}$	8.90 $\times \;10^{2}$
	STD	4.05 $\times \;10^{-1}$	7.35 $\times \;10^{-1}$	5.88 $\times \;10^{0}$		STD	4.95 $\times \;10^{0}$	1.59 $\times \;10^{1}$	6.56 $\times \;10^{1}$
	rank	1	2	3		rank	1	3	2
F8	min	8.13 $\times \;10^{2}$	9.38 $\times \;10^{2}$	8.69 $\times \;10^{2}$	F9	min	9.00 $\times \;10^{2}$	9.02 $\times \;10^{2}$	1.23 $\times \;10^{3}$
	max	8.42 $\times \;10^{2}$	1.02 $\times \;10^{3}$	1.04 $\times \;10^{3}$		max	9.73 $\times \;10^{2}$	1.61 $\times \;10^{3}$	4.19 $\times \;10^{3}$
	avg	8.24 $\times \;10^{2}$	9.89 $\times \;10^{2}$	9.31 $\times \;10^{2}$		avg	9.14 $\times \;10^{2}$	1.00 $\times \;10^{3}$	2.39 $\times \;10^{3}$
	STD	7.35 $\times \;10^{0}$	1.70 $\times \;10^{1}$	4.07 $\times \;10^{1}$		STD	2.07 $\times \;10^{1}$	1.52 $\times \;10^{2}$	8.57 $\times \;10^{2}$
	rank	1	3	2		rank	1	2	3
F10	min	4.61 $\times \;10^{3}$	6.14 $\times \;10^{3}$	3.40 $\times \;10^{3}$	F11	min	1.23 $\times \;10^{3}$	1.16 $\times \;10^{3}$	1.12 $\times \;10^{3}$
	max	5.32 $\times \;10^{3}$	7.82 $\times \;10^{3}$	6.21 $\times \;10^{3}$		max	9.04 $\times \;10^{3}$	1.29 $\times \;10^{3}$	1.26 $\times \;10^{3}$
	avg	4.95 $\times \;10^{3}$	6.87 $\times \;10^{3}$	4.93 $\times \;10^{3}$		avg	3.51 $\times \;10^{3}$	1.23 $\times \;10^{3}$	1.16 $\times \;10^{3}$
	STD	1.74 $\times \;10^{2}$	3.87 $\times \;10^{2}$	8.30 $\times \;10^{2}$		STD	1.66 $\times \;10^{3}$	2.84 $\times \;10^{1}$	3.07 $\times \;10^{1}$
	rank	2	3	1		rank	3	2	1
F12	min	4.04 $\times \;10^{4}$	1.18 $\times \;10^{4}$	3.41 $\times \;10^{4}$	F13	min	3.27 $\times \;10^{3}$	1.60 $\times \;10^{3}$	1.32 $\times \;10^{3}$
	max	1.51 $\times \;10^{7}$	7.61 $\times \;10^{5}$	1.51 $\times \;10^{6}$		max	5.35 $\times \;10^{4}$	5.56 $\times \;10^{4}$	6.05 $\times \;10^{4}$
	avg	3.76 $\times \;10^{6}$	2.31 $\times \;10^{5}$	4.09 $\times \;10^{5}$		avg	2.63 $\times \;10^{4}$	1.31 $\times \;10^{4}$	1.52 $\times \;10^{4}$
	STD	3.01 $\times \;10^{6}$	1.73 $\times \;10^{5}$	4.14 $\times \;10^{5}$		STD	1.24 $\times \;10^{4}$	1.45 $\times \;10^{4}$	1.47 $\times \;10^{4}$
	rank	3	1	2		rank	3	1	2
F14	min	3.30 $\times \;10^{5}$	2.15 $\times \;10^{3}$	2.03 $\times \;10^{3}$	F15	min	2.09 $\times \;10^{3}$	1.88 $\times \;10^{3}$	1.61 $\times \;10^{3}$
	max	2.20 $\times \;10^{6}$	1.25 $\times \;10^{5}$	7.76 $\times \;10^{4}$		max	2.27 $\times \;10^{4}$	1.77 $\times \;10^{5}$	3.31 $\times \;10^{4}$
	avg	1.11 $\times \;10^{6}$	1.83 $\times \;10^{4}$	1.20 $\times \;10^{4}$		avg	7.14 $\times \;10^{3}$	2.42 $\times \;10^{4}$	5.73 $\times \;10^{3}$
	STD	5.12 $\times \;10^{5}$	2.58 $\times \;10^{4}$	1.46 $\times \;10^{4}$		STD	4.47 $\times \;10^{3}$	4.27 $\times \;10^{4}$	6.80 $\times \;10^{3}$
	rank	3	2	1		rank	2	3	1
F16	min	1.86 $\times \;10^{3}$	1.64 $\times \;10^{3}$	1.85 $\times \;10^{3}$	F17	min	1.77 $\times \;10^{3}$	1.77 $\times \;10^{3}$	1.76 $\times \;10^{3}$
	max	3.35 $\times \;10^{3}$	3.26 $\times \;10^{3}$	3.10 $\times \;10^{3}$		max	2.62 $\times \;10^{3}$	2.32 $\times \;10^{3}$	2.50 $\times \;10^{3}$
	avg	2.67 $\times \;10^{3}$	2.45 $\times \;10^{3}$	2.52 $\times \;10^{3}$		avg	2.13 $\times \;10^{3}$	1.91 $\times \;10^{3}$	2.11 $\times \;10^{3}$
	STD	3.27 $\times \;10^{2}$	4.26 $\times \;10^{2}$	3.32 $\times \;10^{2}$		STD	1.85 $\times \;10^{2}$	1.39 $\times \;10^{2}$	2.02 $\times \;10^{2}$
	rank	3	1	2		rank	3	1	2
F18	min	9.29 $\times \;10^{4}$	6.06 $\times \;10^{4}$	3.02 $\times \;10^{4}$	F19	min	2.90 $\times \;10^{3}$	1.97 $\times \;10^{3}$	1.96 $\times \;10^{3}$
	max	2.59 $\times \;10^{6}$	1.27 $\times \;10^{6}$	6.15 $\times \;10^{5}$		max	1.73 $\times \;10^{4}$	3.93 $\times \;10^{5}$	3.36 $\times \;10^{4}$
	avg	9.00 $\times \;10^{5}$	3.83 $\times \;10^{5}$	1.56 $\times \;10^{5}$		avg	6.61 $\times \;10^{3}$	3.11 $\times \;10^{4}$	7.98 $\times \;10^{3}$
	STD	7.58 $\times \;10^{5}$	2.98 $\times \;10^{5}$	1.23 $\times \;10^{5}$		STD	3.44 $\times \;10^{3}$	7.56 $\times \;10^{4}$	6.88 $\times \;10^{3}$
	rank	3	2	1		rank	1	3	2
F20	min	2.65 $\times \;10^{3}$	2.06 $\times \;10^{3}$	2.16 $\times \;10^{3}$	F21	min	2.31 $\times \;10^{3}$	2.45 $\times \;10^{3}$	2.34 $\times \;10^{3}$
	max	3.12 $\times \;10^{3}$	2.72 $\times \;10^{3}$	2.67 $\times \;10^{3}$		max	2.35 $\times \;10^{3}$	2.51 $\times \;10^{3}$	2.43 $\times \;10^{3}$
	avg	2.89 $\times \;10^{3}$	2.34 $\times \;10^{3}$	2.45 $\times \;10^{3}$		avg	2.33 $\times \;10^{3}$	2.48 $\times \;10^{3}$	2.38 $\times \;10^{3}$
	STD	1.60 $\times \;10^{2}$	1.65 $\times \;10^{2}$	1.23 $\times \;10^{2}$		STD	9.82 $\times \;10^{0}$	1.74 $\times \;10^{1}$	2.34 $\times \;10^{1}$
	rank	3	1	2		rank	1	3	2
F22	min	2.30 $\times \;10^{3}$	2.30 $\times \;10^{3}$	2.30 $\times \;10^{3}$	F23	min	2.66 $\times \;10^{3}$	2.81 $\times \;10^{3}$	2.70 $\times \;10^{3}$
	max	2.30 $\times \;10^{3}$	2.31 $\times \;10^{3}$	5.28 $\times \;10^{3}$		max	2.73 $\times \;10^{3}$	2.88 $\times \;10^{3}$	2.82 $\times \;10^{3}$
	avg	2.30 $\times \;10^{3}$	2.30 $\times \;10^{3}$	2.40 $\times \;10^{3}$		avg	2.70 $\times \;10^{3}$	2.84 $\times \;10^{3}$	2.76 $\times \;10^{3}$
	STD	6.23 $\times \;10^{-1}$	1.62 $\times \;10^{0}$	5.44 $\times \;10^{2}$		STD	1.73 $\times \;10^{1}$	1.65 $\times \;10^{1}$	2.71 $\times \;10^{1}$
	rank	1	2	3		rank	1	3	2
F24	min	2.84 $\times \;10^{3}$	2.98 $\times \;10^{3}$	2.87 $\times \;10^{3}$	F25	min	2.89 $\times \;10^{3}$	2.88 $\times \;10^{3}$	2.88 $\times \;10^{3}$
	max	2.88 $\times \;10^{3}$	3.06 $\times \;10^{3}$	3.01 $\times \;10^{3}$		max	3.00 $\times \;10^{3}$	2.94 $\times \;10^{3}$	2.94 $\times \;10^{3}$
	avg	2.85 $\times \;10^{3}$	3.03 $\times \;10^{3}$	2.93 $\times \;10^{3}$		avg	2.93 $\times \;10^{3}$	2.89 $\times \;10^{3}$	2.89 $\times \;10^{3}$
	STD	1.24 $\times \;10^{1}$	1.67 $\times \;10^{1}$	3.36 $\times \;10^{1}$		STD	2.69 $\times \;10^{1}$	1.46 $\times \;10^{1}$	1.33 $\times \;10^{1}$
	rank	1	3	2		rank	3	2	1
F26	min	2.80 $\times \;10^{3}$	4.99 $\times \;10^{3}$	2.80 $\times \;10^{3}$	F27	min	3.20 $\times \;10^{3}$	3.19 $\times \;10^{3}$	3.22 $\times \;10^{3}$
	max	5.08 $\times \;10^{3}$	5.81 $\times \;10^{3}$	7.39 $\times \;10^{3}$		max	3.27 $\times \;10^{3}$	3.27 $\times \;10^{3}$	3.27 $\times \;10^{3}$
	avg	4.20 $\times \;10^{3}$	5.51 $\times \;10^{3}$	4.92 $\times \;10^{3}$		avg	3.24 $\times \;10^{3}$	3.22 $\times \;10^{3}$	3.25 $\times \;10^{3}$
	STD	5.25 $\times \;10^{2}$	2.16 $\times \;10^{2}$	1.39 $\times \;10^{3}$		STD	1.33 $\times \;10^{1}$	1.69 $\times \;10^{1}$	1.56 $\times \;10^{1}$
	rank	1	3	2		rank	2	1	3
F28	min	3.27 $\times \;10^{3}$	3.11 $\times \;10^{3}$	3.19 $\times \;10^{3}$	F29	min	3.51 $\times \;10^{3}$	3.48 $\times \;10^{3}$	3.62 $\times \;10^{3}$
	max	3.42 $\times \;10^{3}$	3.26 $\times \;10^{3}$	3.26 $\times \;10^{3}$		max	4.30 $\times \;10^{3}$	4.12 $\times \;10^{3}$	4.32 $\times \;10^{3}$
	avg	3.31 $\times \;10^{3}$	3.20 $\times \;10^{3}$	3.21 $\times \;10^{3}$		avg	3.87 $\times \;10^{3}$	3.77 $\times \;10^{3}$	3.88 $\times \;10^{3}$
	STD	2.68 $\times \;10^{1}$	3.06 $\times \;10^{1}$	1.95 $\times \;10^{1}$		STD	1.83 $\times \;10^{2}$	1.75 $\times \;10^{2}$	1.71 $\times \;10^{2}$
	rank	3	1	2		rank	2	1	3
F30	min	1.70 $\times \;10^{4}$	5.78 $\times \;10^{3}$	6.25 $\times \;10^{3}$					
	max	2.59 $\times \;10^{6}$	1.95 $\times \;10^{5}$	1.81 $\times \;10^{4}$					
	avg	3.14 $\times \;10^{5}$	3.40 $\times \;10^{4}$	1.03 $\times \;10^{4}$					
	STD	4.80 $\times \;10^{5}$	4.79 $\times \;10^{4}$	2.54 $\times \;10^{3}$					

**Table 7:** Statistical outcomes comparing mAEFA against other metaheuristics applied to speed reducer design.

Algorithms	Rank	Best	Worst	Mean	STD
mAEFA	1	2999.125	3276.964	3081.04	96.0022
AEFA	2	2998.704	3357.957	3161.609	125.7413
LCA	4	3084.932	3394.719	3217.231	81.89584
CHIO	3	3028.974	3438.588	3161.642	81.68969
GOA	8	3034.447	8843.277	4425.907	1503.485
GSA	5	3149.597	3818.236	3443.538	180.2837
POS	6	2993.701	5905.984	3487.556	687.8122
SAO	7	3230.902	8071.254	4153.676	956.0172

**Table 8:** Results of mAEFA versus other metaheuristics on speed reducer design.

	Best	z1	z2	z3	z4	z5	z6	z7
mAEFA	2999.125	3.497593	0.7	17	7.342937	7.943631	3.350136	5.285752
AEFA	2998.704	3.49759	0.7	17	7.627354	7.805211	3.350677	5.285705
LCA	3084.932	3.483299	0.70015	17.05791	8.058002	8.247121	3.452092	5.311111
CHIO	3028.974	3.497172	0.7	17	7.51058	7.711363	3.44856	5.294154
GOA	3034.447	3.571421	0.701486	17	7.649347	7.714521	3.353149	5.288426
GSA	3149.597	3.592483	0.711502	17.12052	7.344929	7.683132	3.454113	5.282249
POS	2993.701	3.49765	0.7	17	7.3	7.712778	3.350306	5.28539
SAO	3230.902	3.6	2.6	3.565453	2.84373	2.951027	3.314187	2.715053

**Table 9:** Statistical results of mAEFA versus other metaheuristics on pressure vessel design problem.

Measures	Best	Worst	Mean	STD	Rank
mAEFA	6412.152	15600.59	8646.057	1887.591	2
AEFA	6769.697	18818.75	9415.145	2473.888	3
LCA	6690.927	9928.012	8388.067	900.3348	1
CHIO	7381.037	13270.55	9741.683	1611.388	4
GOA	7411.902	40809.83	16388.75	8315.391	8
GSA	6390.632	40404.85	15418.54	7878.361	7
POS	6266.019	14690.99	10816.18	2322.216	5
SAO	7710.968	23390.18	13266.08	3334.069	6

**Table 10:** Statistical results of mAEFA compared with other algorithms on pressure vessel design problem.

	Best	z1	z2	z3	z4
mAEFA	6412.152	0.824149	0.407546	42.89068	185.7112
AEFA	6769.697	0.902527	0.445849	46.93715	146.8159
LCA	6690.927	0.838483	0.395264	44.07847	155.5208
CHIO	7381.037	0.926379	0.602382	42.96878	169.9356
GOA	7411.902	1.083653	0.572748	50	98.84785
GSA	6390.632	0.968216	0.477738	50.30862	98.92202
POS	6266.019	0.924327	0.443053	46.73207	126.5877
SAO	7710.968	2.527902	5.801292	1.559886	5.133647

**Table 11:** Statistical results of mAEFA versus other metaheuristics on multi-product batch plant problem.

Measures	Best	Worst	Mean	STD	Rank
mAEFA	63491.75	98704.83	80183.47	8834.615	1
AEFA	61081.05	101036.2	85863.96	9781.795	2
LCA	81552.61	109366.8	98317.28	6991.52	4
CHIO	74939.34	120685.6	97692.22	8345.171	3
GOA	125170.2	4.71 $\times \;10^{10}$	3.39 $\times \;10^{9}$	1.03 $\times \;10^{10}$	7
GSA	83258.78	157410.5	99175.74	11609.84	5
POS	76198.96	165947.4	112619.2	25070.13	6
SAO	80120.89	6.28 $\times \;10^{10}$	5.12 $\times \;10^{9}$	1.26 $\times \;10^{10}$	8

**Table 12:** Results of mAEFA versus other metaheuristics on multi-product batch plant problem.

	Best	x1	x2	x3	x4	x5	x6	x7	x8	x9	x10
mAEFA	63491.75	0.52857	1.349136	1.036593	1745.955	1756.168	1404.395	19.99933	15.99943	233.0765	136.8181
AEFA	61081.05	0.753475	0.635496	0.530559	1555.817	1613.788	1423.447	20	16	227.9073	140.7617
LCA	81552.61	2.123418	0.523666	0.986531	1431.905	1882.782	1377.451	19.98424	8.146837	259.8943	81.26382
CHIO	74939.34	2.463464	1.862752	1.040579	1004.223	904.7103	781.3551	10.0301	8.258278	136.7337	58.49508
GOA	125170.2	1.638572	3.111043	2.266871	1145.717	1248.944	1296.439	7.316115	15.46341	140.0631	125.618
GSA	83258.78	1.07281	1.987149	1.491329	1548.196	1431.676	1419.545	14.63929	15.93547	270.3076	103.232
POS	76198.96	1.576433	1.501728	1.39028	746.8241	1203.952	864.5001	9.999428	8.189601	131.1378	55.74986
SAO	80120.89	2.324455	2.900101	1.435999	2.209186	1.393715	0.792087	3.341121	1.503093	3.419913	2.639791

**Table 13:** Statistical results of mAEFA versus other metaheuristics on design of industrial refrigeration system.

Measures	Best	Worst	Mean	STD	Rank
mAEFA	5103.195	6845.299	5897.153	1243.806	2
AEFA	5854.164	7098.685	6584.685	1123.588	1
LCA	3846.410	27925.458	10666.124	5210.384	6
CHIO	2093.402	35616.788	19387.112	9003.481	7
GOA	8716.021	65297.497	9506.237	14314.970	5
GSA	7799.196	10860.561	4167.086	2660.564	3
POS	6053.37	146 $\times \;10^{8}$	5008.193	26683.805	4
SAO	4246.044	476 $\times \;10^{9}$	788 $\times \;10^{8}$	926 $\times \;10^{8}$	8

**Table 14:** Results of mAEFA versus other metaheuristics on design of industrial refrigeration system.

	mAEFA	AEFA	LCA	CHIO	GOA	GSA	POS	SAO
x1	0.001	0.001	0.353413	0.001	0.001	0.143356	0.001	4.056261
x2	0.001	0.001	0.740792	0.001	0.116401	0.954376	0.001	3.897615
x3	2.936496	5	0.507325	0.172527	3.365744	2.404949	0.001	3.83318
x4	3.167783	0.001	4.210235	3.26012	0.01427	3.091417	0.582783	0.341573
x5	1.65173	5	1.659602	2.89829	2.575789	3.735812	0.001	0.159483
x6	4.565306	0.001	4.517057	1.692646	1.660094	1.984339	0.001	4.162318
x7	2.304624	5	1.896705	2.22803	2.992306	3.061548	1.667485	3.263213
x8	3.539738	5	2.371443	1.740014	3.971143	2.334031	2.171946	3.984312
x9	3.060001	5	4.956327	1.025065	4.629864	2.111681	4.99986	0.914673
x10	4.983777	2.04176	4.226494	5	4.642211	4.546502	1.999999	5
x11	2.626728	0.001	3.40886	3.963239	0.001	3.387399	0.001	0.729443
x12	0.06055	0.001	0.135978	0.205029	0.001	0.07326	0.001	4.060669
x13	0.068226	0.001	0.122193	0.120476	0.001	0.065854	0.001	4.240338
x14	0.797011	0.001	1.593305	3.023032	0.001	1.122458	0.010963	5
best	5103.195	5854.164	3846.410	2093.402	8716.021	779919.6	6053.37	14246.044

## Data Availability

Data are available on request.
